# Multiscale characterization of an inflammatory macrophage state associated with an air pollutant–prioritized prognostic signature in gastric cancer

**DOI:** 10.3389/fimmu.2026.1951968

**Published:** 2026-09-10

**Authors:** Luming Zhao, Nan Hu, Yimeng Xu, Chenxi Mao, Yidong Hong, Jingzhou Zhang, Kangjie Zhou, Fenglei Wu

**Affiliations:** 1 Department of Oncology, The Affiliated Lianyungang Hospital of Xuzhou Medical University/The First People’s Hospital of Lianyungang, Lianyungang, China; 2 Lianyungang Clinical College of Nanjing Medical University/The First People's Hospital of Lianyungang, Lianyungang, China

**Keywords:** air pollutants, gastric cancer, machine learning, molecular dynamics, network toxicology, spatial transcriptomic analysis

## Abstract

**Background:**

Air pollutants are implicated in carcinogenesis, but their relevance to gastric cancer (GC) across prognosis, tumor organization and cell behavior remains unclear. We integrated evidence to evaluate pollutant-prioritized GC candidates.

**Methods:**

Seven pollutants were integrated with GeneCards/OMIM GC genes using toxicology and candidate-prioritization resources. Robustness analyses used alternative disease definitions, Fisher’s tests, 5,000 degree- and annotation-matched null sets, enrichment and protein–protein interaction networks. TCGA-STAD supported nested evaluation of 117 survival pipelines and a five-gene neural network, with locked external testing in GSE84437 and GSE54129. Single-cell, donor-aware macrophage, CellChat, Monocle2, multiplex immunofluorescence and cell2location-guided Visium analyses resolved tissue context. Docking, 100-ns molecular dynamics and bibliometrics examined GSK-3β-centered hypotheses. AGS cells underwent benzene/toluene dose screening followed by CCK-8, colony formation, Transwell and wound-healing assays.

**Results:**

Among 176 genes, 63 overlapped with GC beyond matched-null expectations (empirical P = 0.012), with enrichment in inflammation, hypoxia, apoptosis, metabolism and carcinogenesis. APRGSig (DNMT1, GSK3B, HNF4A, MMP9 and TERT) remained associated with overall survival after age/T/N/M adjustment (HR per 1-SD increase, 2.31; 95% CI, 1.98–2.71) and achieved a locked external C-index of 0.701; exploratory GC-ANN achieved an external AUC of 0.962. Score-defined APRGSig-high macrophages showed broader predicted crosstalk, whereas donor-aware analysis identified an inflammatory/stress-associated MALAT1-high state. Multiplex immunofluorescence provided representative compartment-level localization of APRGSig-encoded proteins; four Visium sections showed directionally consistent positive spatial concordance between APRGSig scores and inferred MALAT1-high abundance. Formal GSK-3β–benzene/toluene models yielded Vina scores of −7.1/−7.4 kcal/mol and remained numerically persistent over 100 ns; bibliometrics of 311 publications highlighted proliferation, invasion and metastasis. At 2.5 mM benzene and 20 μM toluene, both exposures increased AGS clonogenic capacity, Matrigel traversal and wound closure (all P < 0.05).

**Conclusions:**

This framework integrates predictive, tissue-resolved, structural and phenotype-level evidence to prioritize exposure-linked molecular and immune hypotheses in GC.

## 1 Introduction

In recent years, driven by population growth and the rapid pace of urbanization, air pollution has become a formidable global challenge (1). Beyond its profound environmental consequences, mounting evidence implicates air pollution in the etiology and progression of chronic metabolic disorders and malignancies. Major pollutants include fine particulate matter (PM_2.5_), sulfur dioxide (SO_2_), ozone (O_3_), nitrogen oxides (NO_2_ and NO_x_), carbon monoxide (CO), and volatile organic compounds (VOCs) (2). These agents are thought to perturb immune homeostasis—via mechanisms such as oxidative stress, sustained inflammation, and endocrine disruption—thereby precipitating a broad spectrum of disease states (3). The seven pollutants (benzene, SO_2_, NO, CO, NO_2_, toluene, O_3_) were selected because they are ubiquitous, regulated exposures with documented links to oxidative stress, chronic inflammation (4), and chemical-carcinogenesis pathways relevant to GC biology; we cite recent public-health syntheses to contextualize their global relevance while not inferring GC-specific causality (5). We emphasize that the present study does not quantify individual-level exposure. Accordingly, throughout the manuscript “pollutant-related” refers to a pollutant-prioritized molecular program assembled from public resources and in-silico prioritization, rather than measured exposure response. In addition, for highly reactive gases (e.g., O_3_, NO/NO_2_, CO, SO_2_), the framework is intended to prioritize biologically plausible downstream nodes (e.g., oxidative-stress and inflammatory signaling) rather than to assert direct ligand–protein binding *in vivo*.

Gastric cancer (GC) constitutes the fifth most frequently diagnosed malignancy worldwide and ranks among the four leading causes of cancer-related mortality (6). According to the International Agency for Research on Cancer (IARC) 2022 report, GC remains a formidable global public-health challenge. Accounting for approximately 4.9% of all newly diagnosed cancer cases, GC underscores a persistently high incidence on a global scale. Furthermore, it is responsible for about 6.8% of all cancer-related deaths, making it one of the most lethal cancers (7). Nevertheless, the impact of environmental factors, lifestyle, and diet on gastric cancer remains incompletely understood. Although several studies have indicated that long-term exposure to air pollutants may increase the risk of gastric cancer (8), the specific molecular mechanisms involved are still unclear.

In recent years, advances in bioinformatics have provided novel tools for dissecting the complex interactions between environmental exposures and disease. Network toxicology—by integrating multi-omics datasets with network-based analyses—has emerged as a critical approach for identifying molecular targets and pathways involved in pathogenesis. Moreover, machine learning algorithms have demonstrated considerable potential for disease prediction and the high-precision identification of biomarkers.

**Figure 1:**
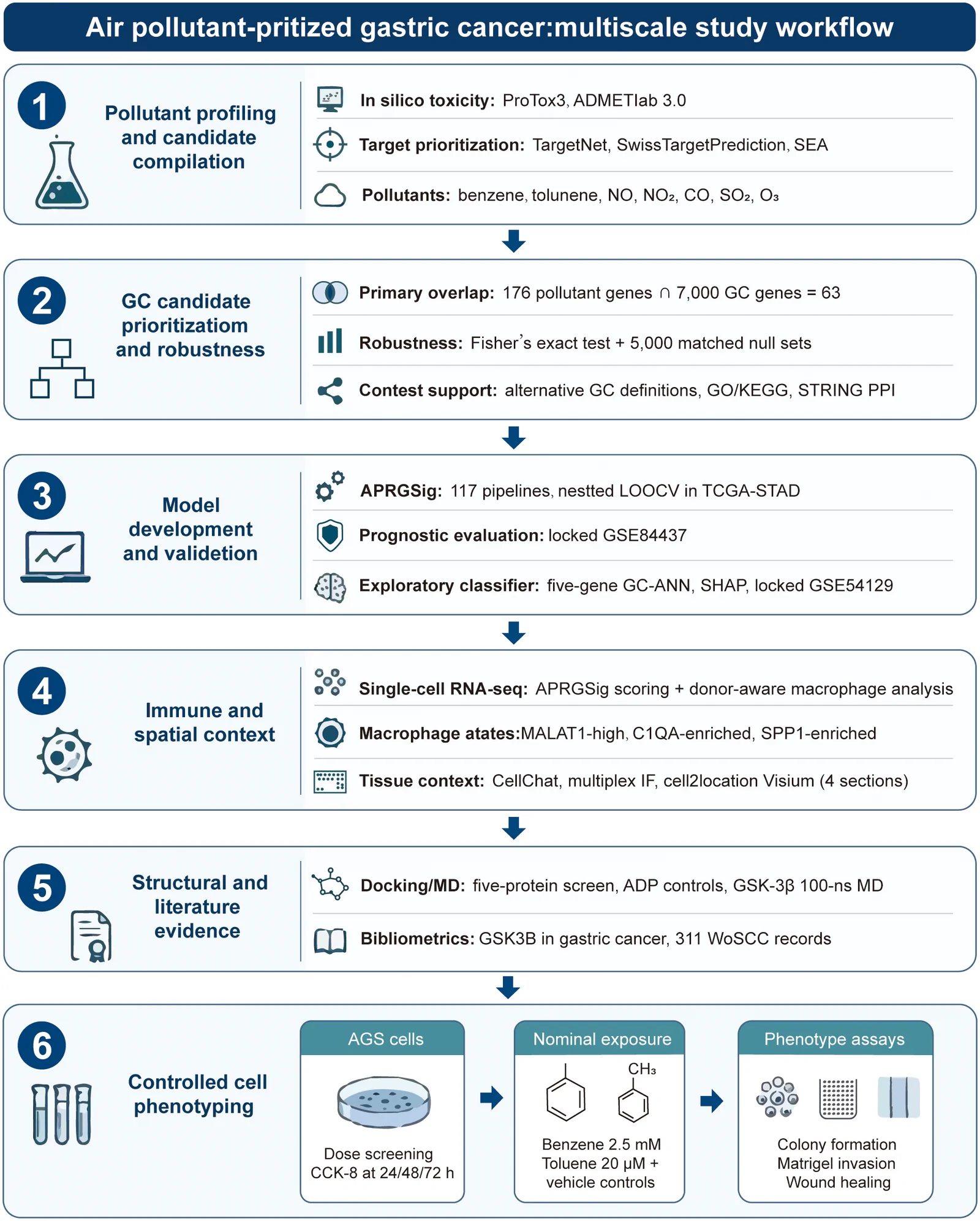
Study design overview.

In this study, we investigated seven prevalent air pollutants—benzene, sulfur dioxide (SO_2_), nitric oxide (NO), carbon monoxide (CO), nitrogen dioxide (NO_2_), toluene and ozone (O_3_)—through a cross-scale framework integrating network toxicology, machine learning with SHAP interpretation, an artificial neural network, single-cell and spatial transcriptomics, multiplex immunofluorescence, molecular docking and dynamics, bibliometrics, and controlled *in vitro* experiments (**Figure 1**). Pollutant-prioritized candidates were integrated with bulk transcriptomic and clinical cohorts to derive externally evaluated prognostic and tissue-classification models, then localized across tumor microenvironmental states and tissue compartments. Structural and bibliometric analyses prioritized GSK3B-centered hypotheses for benzene and toluene, while controlled exposure assays quantified their effects on AGS cell viability, clonogenicity, invasion and migration. By progressing from candidate discovery and external evaluation to tissue localization, structural prioritization, and focused cellular phenotyping, the study integrates complementary molecular, spatial, and phenotype-level evidence within a multiscale framework for GC. The controlled AGS experiments provided a focused exploratory phenotypic assessment of the benzene/toluene branch in a defined gastric cancer cellular context.

## 2 Methods and materials

### 2.1 Evaluation of the toxicological profiles of airborne contaminants

Chemical structures and associated molecular information for the seven selected air pollutants were obtained from the PubChem database (https://pubchem.ncbi.nlm.nih.gov). Carcinogenic potential was subsequently assessed using the ADMETlab 3.0 platform (https://admetlab3.scbdd.com) and the ProTox3 database (https://tox.charite.de/protox3).

### 2.2 Compilation of air pollutant-prioritized candidate genes

Candidate genes were retrieved separately for benzene, toluene, nitric oxide (NO), nitrogen dioxide (NO_2_), carbon monoxide (CO), sulfur dioxide (SO_2_), and ozone (O_3_) using TargetNet, SwissTargetPrediction, and the Similarity Ensemble Approach. Pollutant-specific gene lists were retained before merging to preserve source provenance, and duplicate genes were removed to generate a non-redundant candidate set for downstream analyses. Because conventional ligand-based target-prediction frameworks are less suited to highly reactive or inorganic gases, candidates identified for NO, NO_2_, CO, SO_2_, and O_3_ were interpreted as exploratory downstream molecular priorities rather than evidence of direct ligand–protein binding. Structural and experimental analyses therefore focused on benzene and toluene.

### 2.3 Collection of genes implicated in gastric carcinogenesis

GeneCards (v5.23; accessed March 5, 2025) and Online Mendelian Inheritance in Man (OMIM; database snapshot accessed March 5, 2025) were queried using “gastric cancer.” For the primary disease-gene definition, GeneCards genes with relevance scores ≥0.67 and the top 25% of OMIM entries were retained, followed by gene-symbol standardization, merging, and deduplication (9). To assess robustness to disease-gene definition, two alternative sets were analyzed independently: a broader OMIM-only set derived from the same query and TCGA-STAD tumor-versus-non-tumor differentially expressed genes (FDR <0.05; |log_2_ fold change| ≥1).

Enrichment was evaluated using two-sided Fisher’s exact tests against a prespecified universe of 25,000 eligible genes from the HGNC Complete Set (January 2025 snapshot; accessed March 11, 2025). Eligible genes required an approved HGNC symbol, a valid NCBI Gene identifier, one-to-one identifier mapping, and representation in STRING v12.0 or GO 2025-02-06. Pseudogenes, withdrawn or superseded records, invalid NCBI Gene records, and duplicate mappings were excluded.

For matched-null testing, 5,000 random 176-gene sets were generated by jointly matching STRING degree and GO annotation count. STRING degree was defined as the number of unique interaction partners at a combined score ≥0.400, and GO annotation count as the number of unique assigned GO terms. Zero values formed separate bins, whereas positive values were divided into quartiles, yielding 25 joint strata. One background gene was sampled without replacement for each observed gene from the corresponding stratum, excluding the 176 observed genes. Missing STRING or GO annotations were assigned zero. Sampling used seed 12345678, and empirical P values were calculated as (k+1)/(5000 + 1).

### 2.4 Gene Ontology and KEGG pathway enrichment analysis

GO and KEGG enrichment analyses of the shared pollutant- and gastric cancer-associated candidates were performed using DAVID. Gene Ontology biological process, molecular function, and cellular component terms, together with KEGG pathways, were evaluated, and representative enrichment results were visualized using https://bioinformatics.com.cn (accessed March 11, 2025). KEGG annotations were cited according to official guidance (10).

### 2.5 PPI analysis and network prioritization of shared candidates

Protein–protein interactions among shared candidates were retrieved from the Homo sapiens STRING v12.0 functional-association network using a combined confidence score ≥0.400. The resulting network was visualized in Cytoscape 3.10.3, and degree centrality was used to summarize network connectivity and prioritize highly connected candidates.

### 2.6 Public transcriptomic dataset selection and bulk cohort-specific preprocessing

Publicly available transcriptomic datasets were incorporated to provide complementary evidence across external model evaluation, single-cell characterization, and spatial analysis of gastric cancer. TCGA-STAD served as the development cohort; GSE84437 and GSE54129 supported external prognostic and tumor-versus-non-tumor evaluation, respectively, while GSE163558 and GSE251950 supported single-cell and spatial analyses. For TCGA-STAD, FPKM values were converted to TPM, log_2_(TPM + 1)-transformed, and mapped to HGNC symbols. For GSE84437, quantile-normalized microarray expression was log_2_-transformed, mapped from probes to gene symbols, and gene-wise standardized before applying the locked APRGSig coefficients and TCGA-derived median cutoff unchanged. Cases lacking valid overall-survival time or event status were excluded without outcome imputation (Supplementary Methods S1). Raw GSE54129 CEL files underwent RMA normalization and gene-level probe consolidation. Bulk cohorts were processed independently without cross-cohort ComBat correction, maintaining their predefined development and external-evaluation roles (**Supplementary Table 1**).

### 2.7 Development and validation of the APRGSig prognostic model

A gastric cancer prognostic signature (APRGSig) was developed from pollutant-prioritized candidate genes by evaluating 117 prespecified survival pipelines spanning Lasso, Ridge, Elastic Net, StepCox, survivalSVM, CoxBoost, SuperPC, plsRcox, RSF, and GBM (11). Model development and selection were confined to TCGA-STAD using nested leave-one-out cross-validation (LOOCV). Within each outer-training partition, preprocessing, feature screening, hyperparameter tuning, model fitting, and coefficient estimation were repeated *de novo*, with learned transformations applied unchanged to the held-out patient. Pipelines were ranked by aggregated out-of-fold Harrell C-indices, with a prespecified parsimony rule applied only to exact ties. Within the selected RSF–LASSO workflow, RSF retained the 15 highest-ranking genes, whereas LASSO-Cox (α = 1) retained nonzero features at λmin selected by 10-fold inner cross-validation; the five genes retained by both procedures defined APRGSig. The workflow was refitted in full TCGA-STAD to estimate final coefficients and the fixed median cutoff before locked evaluation in GSE84437. Candidate-pipeline performance across cohorts was subsequently summarized for post-selection descriptive comparison, while model selection remained based exclusively on TCGA-STAD internal validation.

### 2.8 Evaluation of prognostic performance

Prognostic performance was assessed in TCGA-STAD and independently in GSE84437. Internal discrimination used nested-LOOCV held-out predictions to estimate Harrell’s C-index and 95% confidence interval, while full-cohort refitting and bootstrap repetition of the complete development workflow provided apparent and optimism-corrected estimates. The locked model was applied to GSE84437 without feature reselection, coefficient re-estimation, model updating, or cutoff recalibration. Time-dependent ROC curves evaluated 1-, 3-, and 5-year overall survival, while calibration intercepts and slopes and time-specific and integrated Brier scores assessed calibration and prediction error. Prediction-error curves used TCGA-STAD out-of-fold and GSE84437 external predictions, with Kaplan–Meier as the reference. Risk groups were defined using the fixed TCGA-STAD median cutoff and compared by log-rank test. In GSE84437, the proportional-hazards assumption for APRGSig was evaluated using scaled Schoenfeld residuals, with P>0.05 indicating no evidence of violation.

### 2.9 Artificial neural network modeling and validation

An exploratory multilayer perceptron classifier (GC-ANN) integrated the prespecified DNMT1, TERT, GSK3B, MMP9, and HNF4A expression profiles to distinguish gastric cancer from non-tumor gastric tissue. Model development and internal validation were restricted to TCGA-STAD using patient-grouped, class-stratified five-fold outer cross-validation repeated 10 times, with five-fold inner cross-validation for model tuning. Within each training partition, gene-wise scaling and inverse-frequency class weighting were estimated *de novo*. Hidden-layer size (1, 2, 3, 4, 5, 6, or 8 neurons) and L2 weight decay (0–10⁻¹) were optimized by inner-fold AUC. The finalized network comprised five input nodes, one five-neuron logistic hidden layer, and two softmax output nodes (12). BFGS optimization was used with a maximum of 2,000 iterations; convergence was controlled by the optimizer stopping criterion. Random initialization used master seed 12345678 with deterministic fold- and restart-specific offsets, with three restarts for selected fits. Aggregated outer-fold probabilities provided internal predictions, and their Youden-index threshold (0.825) was locked before one-time evaluation in GSE54129. Bootstrap resampling assessed optimism. GC-ANN was benchmarked against logistic, LASSO-logistic, random-forest, and single-gene models using matched resampling, with discrimination, calibration, classification, and prediction-error metrics evaluated comprehensively.

### 2.10 Model interpretation

The finalized GC-ANN fitted to the complete TCGA-STAD development cohort was interpreted using SHapley Additive exPlanations (SHAP) on the predicted-probability scale, with the development cohort serving as the reference distribution. Gene-specific SHAP values decomposed each prediction into contributions from the five input features relative to the model reference value. Mean absolute SHAP values summarized global predictive contribution, while summary and dependence plots characterized the direction and expression-dependent patterns of feature effects. To illustrate sample-level decision structure, a correctly classified tumor sample was selected for the force plot, showing how individual contributions combined to form the final prediction without affecting global ranking. SHAP values were therefore interpreted as model-specific explanations of predictive behavior, with correlated predictors understood as sharing predictive attribution.

### 2.11 Expression patterns and prognostic implications of APRGSig genes

GEPIA was used to compare the mRNA expression of DNMT1, GSK3B, HNF4A, MMP9, and TERT between TCGA-STAD gastric tumor samples (n = 408) and TCGA-STAD adjacent non-tumor gastric tissues (n = 36). GTEx normal tissues were not included in this comparison. Overall-survival associations were evaluated using Kaplan–Meier Plotter with the indicated gastric-cancer microarray probe set for each gene. The platform’s “auto select best cutoff” option was applied separately to each gene, with candidate expression cutoffs evaluated between the lower and upper quartiles. Hazard ratios and 95% confidence intervals were estimated using univariate Cox proportional-hazards models, and survival distributions were compared using the log-rank test. To contextualize the molecular background of the AGS model, baseline expression of DNMT1, GSK3B, HNF4A, MMP9, and TERT across gastric cancer cell lines was additionally examined using ShinyTHOR. In the Multiple-Analyte Gene Expression module, cell lines were filtered by organ (stomach), histology (carcinoma), and pathology (All), and the five genes were visualized using the platform-generated scaled-expression heatmap with hierarchical clustering.

### 2.12 Construction and evaluation of the prognostic nomogram

Using the survival package (version 3.8.3), Cox proportional-hazards models evaluated overall survival. Age was categorized as <60 or ≥60 years; sex and pathological T, N, and M stages were treated as categorical variables, and the APRGSig risk score was modeled continuously, with hazard ratios reported per 1-SD increase. Univariate analyses were performed first, followed by a multivariable model including age group, pathological T, N, and M stages, and APRGSig risk score. The multivariable model was used to construct a nomogram with rms (version 7.0.0) for 1-, 2-, 3-, and 5-year overall survival, with calibration assessed at each time point.

### 2.13 Single-cell RNA-sequencing analysis

Single-cell RNA-sequencing data from GSE163558 were analyzed using Seurat (13, 14). After exclusion of the adjacent non-tumoral sample NT1, nine gastric cancer specimens from six patients were included: three primary tumors, two liver metastases, two lymph-node metastases, one ovarian metastasis, and one peritoneal metastasis. Repeated specimens from the same patients retained shared donor identities for downstream donor-aware analyses. Cells with 200–6,000 detected genes and <20% mitochondrial transcripts were retained; UMI counts were assessed according to sample-specific distributions, and genes detected in ≥3 cells were included. Data were log-normalized and batch-corrected with Harmony. The 2,000 most variable genes were used for principal-component analysis and graph-based clustering at resolution 0.4. Cell identities were assigned using canonical markers and cluster-specific differential-expression signatures. Candidate-gene distributions across annotated cell types and Gene Ontology enrichment were subsequently evaluated.

### 2.14 Multiplex immunofluorescence staining

Archived, de-identified gastric adenocarcinoma specimens were analyzed in accordance with the Declaration of Helsinki under approval from the Ethics Committee of the Affiliated Lianyungang Hospital of Xuzhou Medical University/The First People’s Hospital of Lianyungang City (KY-20251120003-01); written informed consent was waived. Formalin-fixed, paraffin-embedded tumors from four patients undergoing radical gastrectomy were sectioned at 5 μm. Four panels were examined: GSK3B/CD31/Pan-CK/CD68/FAP, HNF4A/MMP9/Pan-CK/CD68, DNMT1/CD31/Pan-CK/CD68/FAP, and TERT/Pan-CK. Antibodies were GSK3B (Abcam, ab32391, Y174, 1:200), DNMT1 (ab188453, EPR18453, 1:100), HNF4A (ab181604, EPR16885, 1:2,000), MMP9 (ab76003, EP1254, 1:100), TERT (Proteintech, 84636-1-RR, 241074H2, 1:500), Pan-CK (Abcam, ab86734, AE1/AE3 + 5D3, 1:200), CD68 (ab955, KP1, 1:3,000), CD31 (ab9498, JC/70A, 1:100), and FAP (ab207178, EPR20021, 1:1,000). Sections were deparaffinized, rehydrated, and microwave-retrieved in citrate or EDTA buffer. Endogenous peroxidase was blocked with 3% H_2_O_2_, followed by serum or BSA blocking. Primary antibodies were incubated overnight at 4 °C, followed by HRP-conjugated secondary antibodies and tyramide signal amplification. Antibody complexes were removed between cycles by microwave citrate treatment. Species- and isotype-matched controls were processed in parallel under identical conditions. After DAPI counterstaining, autofluorescence quenching, and mounting, images were acquired using a Nikon fluorescence microscope and Pannoramic MIDI scanner. Pan-CK, CD68, CD31, and FAP defined epithelial, macrophage-associated, endothelial, and stromal compartments, respectively.

### 2.15 APRGSig scoring and cell–cell communication inference

Single-cell APRGSig scores were calculated from DNMT1, GSK3B, HNF4A, MMP9, and TERT using Seurat AddModuleScore. Relative absolute gene contributions to the macrophage–non-macrophage contrast were defined as the absolute centered gene-specific contrast divided by the summed absolute contrasts. Robustness was evaluated by sequential leave-one-gene-out scoring, rank-based UCell, and 5,000 random five-gene signatures matched for mean expression and detection frequency. Scores were aggregated by patient for confidence intervals, FDR-adjusted comparisons, and empirical significance testing. For CellChat analysis with CellChatDB.human (15), macrophages within each specimen were split at the specimen-specific median APRGSig score into APRGSig-high and APRGSig-low groups and analyzed together with seven non-macrophage lineages. These score-based groups were used only for communication inference and were distinct from transcriptionally defined macrophage states. Communication probabilities were estimated without population-size weighting, excluding groups with <10 cells; incoming/outgoing strengths, pathway probabilities, and representative ligand–receptor pairs were extracted.

### 2.16 Construction of macrophage cell atlas and trajectory analysis

From the annotated dataset, 3,154 macrophages were reprocessed by normalization, highly variable feature selection, batch correction, dimensionality reduction, and graph-based clustering (FindClusters resolution 1.0). Clusters were annotated *post hoc* using differentially expressed genes and canonical myeloid markers, yielding transcriptionally defined macrophage states independently of the APRGSig-high/low median split used for CellChat. APRGSig was recalculated with Seurat AddModuleScore. Patients were treated as the independent biological units; repeated specimens among the nine specimens from six patients were combined within patient before inference. Robustness analyses included gene-level decomposition, leave-one-gene-out scoring, UCell, and 5,000 expression- and detection-matched random signatures. Paired patient-level comparisons evaluated sequencing depth, detected-feature number, mitochondrial-transcript percentage, doublet proportion, and ambient-RNA score. For donor-aware differential expression, raw UMI counts were aggregated within patient and macrophage state. MALAT1-high macrophages were compared with pooled C1QA-enriched and SPP1-enriched macrophages, yielding 12 pseudo-bulk samples from six paired patients. After filterByExpr and TMM normalization, limma–voom used the design ~ patient + macrophage_state with robust empirical Bayes moderation and Benjamini–Hochberg correction. Functional enrichment used significantly upregulated genes against the pseudo-bulk testing universe. To assess dependence on MALAT1, clustering was repeated after excluding MALAT1 from highly variable features, with retention rate, Jaccard index, and adjusted Rand index quantifying concordance. Monocle2 was used for exploratory pseudotemporal ordering and visualization of macrophage-state distributions and selected gene-expression trends (16).

### 2.17 Processing and quantitative analysis of gastric cancer spatial transcriptomic data

Four publicly available 10x Visium sections (GSM7990480, GSM7990479, GSM7990478, and GSM7990475) from GSE251950 were analyzed independently (17). The annotated single-cell RNA-sequencing atlas served as the cell2location reference. Genes with total counts <5 and reference populations comprising <3% of cells were excluded; the expected abundance was set at approximately 5–10 cells per spot and the detection hyperparameter at 200 (18). Cell2location estimated spot-level abundances of major lineages and macrophage states, including MALAT1-high macrophages. Spatial-neighborhood graphs were Leiden-clustered (resolution 0.4), and domains were annotated from transcriptional and inferred-composition profiles. Spot-level APRGSig scores were calculated from DNMT1, GSK3B, HNF4A, MMP9, and TERT. Within each section, global Moran’s I quantified spatial autocorrelation, Spearman correlation assessed spot-level concordance, and bivariate Moran’s I evaluated neighborhood-aware cross-association between APRGSig and inferred MALAT1-high abundance. Permutation P values were Benjamini–Hochberg adjusted. Cell2location outputs were reported as model-inferred spot-level abundance estimates.

### 2.18 Molecular docking

Exploratory molecular docking screened benzene and toluene against the proteins encoded by DNMT1, GSK3B, HNF4A, MMP9, and TERT. Protein Data Bank structures were stripped of crystallographic water molecules, hydrogenated, and converted to docking-compatible formats. PubChem ligands were optimized with the Merck Molecular Force Field and assigned Gasteiger partial charges. AutoDock Vina docked ligands into structurally defined pockets and ranked poses by Vina score. GSK3B underwent focused protocol validation using the chain-A nucleotide-binding pocket of the ADP-bound structure (PDB 1J1C), with crystallographic Mg²^+^ retained. Computational controls comprised redocking the co-crystallized ADP ligand, docking a known GSK3B inhibitor, and docking a physicochemical decoy. Poses were inspected in Discovery Studio 2019 and PyMOL 2.6. The inhibitor and decoy served as positive and negative computational controls, respectively.

### 2.19 Molecular dynamics simulations

Top-ranked AutoDock Vina poses for benzene–target and toluene–target complexes initialized molecular dynamics simulations in GROMACS 2023. Proteins used CHARMM36 and ligands used General AMBER Force Field 2 (GAFF2) parameters. Each complex was placed in a periodic cubic box, solvated with TIP3P water, and treated with particle mesh Ewald electrostatics and a Verlet cutoff for short-range nonbonded interactions. Sequential NVT and NPT equilibration each ran for 100,000 steps (approximately 100 ps; coupling constant, 0.1 ps). Production simulations ran for 100 ns at 310 K and 1 bar to characterize the finite-trajectory behavior of these formal starting models. Trajectories were summarized by root mean square deviation (RMSD), root mean square fluctuation (RMSF), radius of gyration (Rg), and solvent-accessible surface area (SASA). Relative conformational free-energy landscapes used RMSD and Rg as collective coordinates to describe sampled occupancy.

### 2.20 Bibliometric analysis

Among the five APRGSig genes, GSK3B was selected for exploratory bibliometric analysis because it showed a relatively high contribution to the fitted model, while the encoded GSK3B protein yielded comparatively favorable formal docking scores for benzene and toluene within the adopted computational workflow. A comprehensive literature search was conducted in the Web of Science Core Collection (WoSCC) on April 10, 2026 (19). The search strategy combined GSK3B-related terms, including “GSK3B,” “GSK-3β,” “GSK-3 beta,” “GSK3 beta,” “GSK3-beta,” “glycogen synthase kinase 3 beta,” “glycogen synthase kinase-3 beta,” “glycogen synthase kinase 3β,” “glycogen synthase kinase-3β,” “serine/threonine-protein kinase GSK3B,” and “serine/threonine protein kinase GSK3B,” with gastric cancer-related terms, including “gastric cancer,” “gastric cancers,” “cancer, gastric,” “cancers, gastric,” “stomach cancer,” “stomach cancers,” “cancer, stomach,” “cancers, stomach,” “cancer of stomach,” “cancer of the stomach,” “stomach neoplasm,” and “gastric neoplasm.” The search was restricted to Article and Review publications in English. This strategy yielded 311 eligible records, comprising 301 original articles (96.78%) and 10 reviews (3.22%). All records were exported from WoSCC and subsequently analyzed using CiteSpace (20) and VOSviewer (21) to characterize publication dynamics, collaborative networks, intellectual structure, and research hotspots in this field.

### 2.21 Cell culture and chemical exposure

Human AGS gastric adenocarcinoma cells were maintained in RPMI-1640 medium (Gibco) supplemented with 10% fetal bovine serum (FBS; Gibco) at 37 °C in a humidified atmosphere containing 5% CO_2_. Benzene and toluene were administered separately, with the corresponding vehicle-treated cells serving as controls. For concentration screening, AGS cells were exposed to benzene (0, 0.1, 0.5, 1, 2.5, 5, 10, or 20 mM) or toluene (0, 2.5, 5, 10, 20, 40, 80, or 160 μM) for 24, 48, or 72 h. Working concentrations for subsequent phenotypic assays were selected from the concentration screen using relative preservation of cell viability across the 24–72-h interval as the experimental criterion. The selected concentrations were regarded as *in vitro* working conditions.

### 2.22 Cell viability assay

Cell viability was assessed using the Cell Counting Kit-8 assay (CCK-8; TargetMol, C0005). AGS cells were seeded in 96-well plates at approximately 5 × 10³ cells per well and allowed to adhere overnight before exposure to the indicated concentrations of benzene or toluene for 24, 48, or 72 h. At each time point, the exposure medium was removed and replaced with 200 μL of CCK-8 working solution consisting of 20 μL CCK-8 reagent and 180 μL culture medium (10% v/v). Cell-free wells containing the same working solution were included as blanks. Plates were incubated at 37 °C for 30 min, gently shaken for 5 min, and absorbance was measured at 450 nm using a microplate reader. Blank absorbance was subtracted from all measurements before normalization. Cell viability was expressed relative to the corresponding blank-corrected vehicle control, which was defined as 100%.

### 2.23 Colony formation assay

AGS cells were seeded in six-well plates at 1 × 10³ cells per well and allowed to attach for 8–12 h. The cells were subsequently exposed to vehicle, 2.5 mM benzene, or 20 μM toluene for 8 days. Colonies were fixed with methanol, stained with hematoxylin, and counted manually. Colony-forming capacity was compared with that of the corresponding vehicle-treated control.

### 2.24 Transwell invasion assay

Cell invasion was assessed using Matrigel-coated Transwell inserts. AGS cells were resuspended in serum-free medium containing vehicle, 2.5 mM benzene, or 20 μM toluene and seeded into the upper chambers at 5 × 10^4^ cells per insert. Medium supplemented with 10% FBS was added to the lower chambers as a chemoattractant. After 24 h, cells remaining on the upper surface of the membrane were removed with a cotton swab. Cells that had traversed the membrane were fixed, stained with crystal violet, imaged under a microscope, and counted.

### 2.25 Wound-healing assay

AGS cells were cultured in six-well plates until confluent and serum-starved for 24 h. A linear wound was generated using a sterile 200-μL pipette tip, after which the cells were incubated in serum-free medium containing vehicle, 2.5 mM benzene, or 20 μM toluene. Images were acquired at 0 and 24 h using an inverted microscope. Wound areas were measured using ImageJ, and wound closure was calculated as follows: wound closure (%) = (initial wound area − wound area at 24 h)/initial wound area × 100.

### 2.26 Statistical analysis

Cell-based analyses were performed using GraphPad Prism 10.3.1. Each condition included three independent biological experiments, each with three technical replicate wells. Technical replicates were averaged within each experiment, and biological-experiment means were treated as independent observations (n = 3). Data are presented as mean ± SD. CCK-8 data were analyzed separately for benzene and toluene using two-way ANOVA followed by Dunnett’s multiple-comparison test against the corresponding vehicle control within each time point. Colony-formation, Transwell, and wound-healing data were analyzed using one-way ANOVA with Dunnett’s correction. Two-sided adjusted P < 0.05 was considered significant. Benjamini–Hochberg FDR correction was applied to high-dimensional analyses where appropriate.

## 3 Results

### 3.1 In silico toxicological characterization of air pollutants

ADMETlab 3.0 and ProTox3 generated non-zero predicted carcinogenicity scores for all seven compounds, providing comparative model-derived information for computational prioritization (**Table 1**).

**Table 1:** Molecular descriptors and predicted carcinogenicity scores of the seven air pollutants.

Name	SMILES structures	Molecular weight	Predicted carcinogenicity score (ProTox3)	Predicted carcinogenicity score (admetlab3.0)
Benzene	C1=CC=CC=C1	78.21g/mol	0.93	0.968
Toluene	CC1=CC=CC=C1	92.13g/mol	0.89	0.956
NO	[N]=O	30.016g/mol	0.58	0.634
NO_2_	N(=O)[O]	46.005g/mol	0.52	0.975
CO	[C-]#[O+]	28.11g/mol	0.53	1
SO_2_	O=S=O	64.059g/mol	0.61	0.785
O_3_	[O-][O+]=O	47.988g/mol	0.53	0.982

The table summarizes the SMILES structures, molecular weights, and model-derived predicted carcinogenicity scores of the seven air pollutants obtained from ProTox3 and ADMETlab 3.0. These in silico scores were used for comparative computational prioritization and do not constitute classification of the compounds as human carcinogens or epidemiological evidence of carcinogenicity.

### 3.2 Retrieval and curation of air pollutant-prioritized candidate genes

The prediction resources returned 128 candidates for benzene, 149 for toluene, 109 for NO, 102 for NO_2_, 110 for CO, 107 for SO_2_, and 88 for O_3_ (**Supplementary Table 2A**). After merging and deduplication, a non-redundant cross-pollutant union of 176 candidate genes was retained for downstream analysis.

### 3.3 Acquisition of genes correlated with gastric cancer

GeneCards yielded 14,092 gastric cancer-associated genes, of which 6,958 met the prespecified relevance-score cutoff (≥0.67). OMIM returned 288 entries, with the top 72 (25%) retained for the primary disease-gene definition. After gene-symbol harmonization, merging, and deduplication, these resources yielded 7,000 unique gastric cancer-associated genes for downstream candidate prioritization (**Supplementary Table 3**).

### 3.4 Robustness assessment and PPI analysis of shared pollutant–gastric cancer candidates

**Figure 2:**
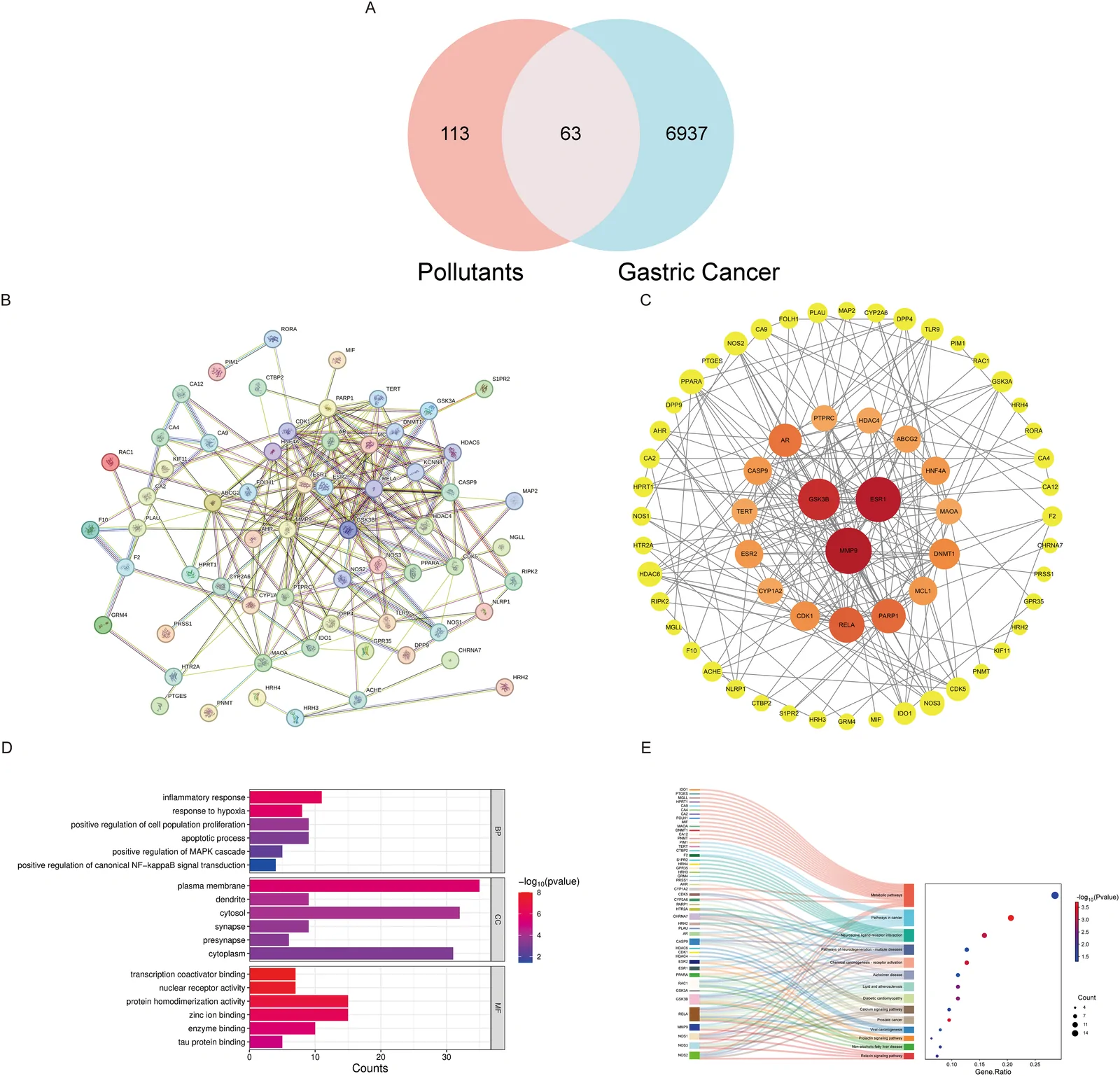
**(A)** Intersection of 176 air pollutant–prioritized candidate genes with 7,000 gastric cancer-associated genes. **(B)** STRING protein–protein interaction network of the shared candidates at a combined-score threshold of ≥0.4. Nodes represent proteins and edges represent STRING-supported functional associations. **(C)** Cytoscape network visualization, with node size and color intensity proportional to degree centrality. **(D)** GO enrichment analysis of the shared candidates. **(E)** KEGG pathway enrichment analysis of the shared candidates. KEGG annotations were obtained from the KEGG database (10).

Intersecting the 176-gene cross-pollutant union with the primary gastric cancer-associated set yielded 63 prioritized candidates (**Supplementary Table 2B**). This overlap exceeded both chance expectation (expected overlap, 49.28 genes; fold enrichment = 1.28; OR = 1.44, 95% CI 1.05–1.96; Fisher’s exact P = 0.0229) and the matched-null distribution (mean = 49.16; 95% interval, 38–61; empirical P = 0.0120; **Supplementary Table 4A**). Consistent enrichment was observed with both alternative disease-gene definitions: the broader OMIM-only set (n = 2,945) yielded 34 overlaps (fold enrichment = 1.64; empirical P = 0.0024), and the TCGA-STAD differential-expression set yielded 40 overlaps (fold enrichment = 1.69; empirical P = 0.0020). Among the 63 primary candidates, 34 were retained by the OMIM-only definition, 28 by TCGA-STAD differential expression, and 23 by both (**Supplementary Table 4B**). All five APRGSig genes were supported by at least two definitions, with DNMT1, HNF4A, MMP9, and TERT supported across all three (**Supplementary Table 4C**). STRING retained all 63 candidates, and degree centrality summarized network connectivity (**Figures 2A–C**).

### 3.5 GO and KEGG pathway–level enrichment profiling

GO enrichment of the 63 shared candidates highlighted inflammatory responses, apoptosis, hypoxia-related processes, cell proliferation, NF-κB signaling, and MAPK activation (**Figure 2D**). KEGG enrichment identified chemical carcinogenesis, pathways in cancer, lipid metabolism and atherosclerosis, relaxin signaling, viral carcinogenesis, insulin resistance, metabolic pathways, and prolactin signaling (**Figure 2E**). Collectively, these enrichment patterns indicated functional convergence on inflammatory, apoptotic, hypoxia-related, metabolic, and carcinogenesis-associated processes.

### 3.6 Construction and validation of the APRGSig prognostic model

**Figure 3:**
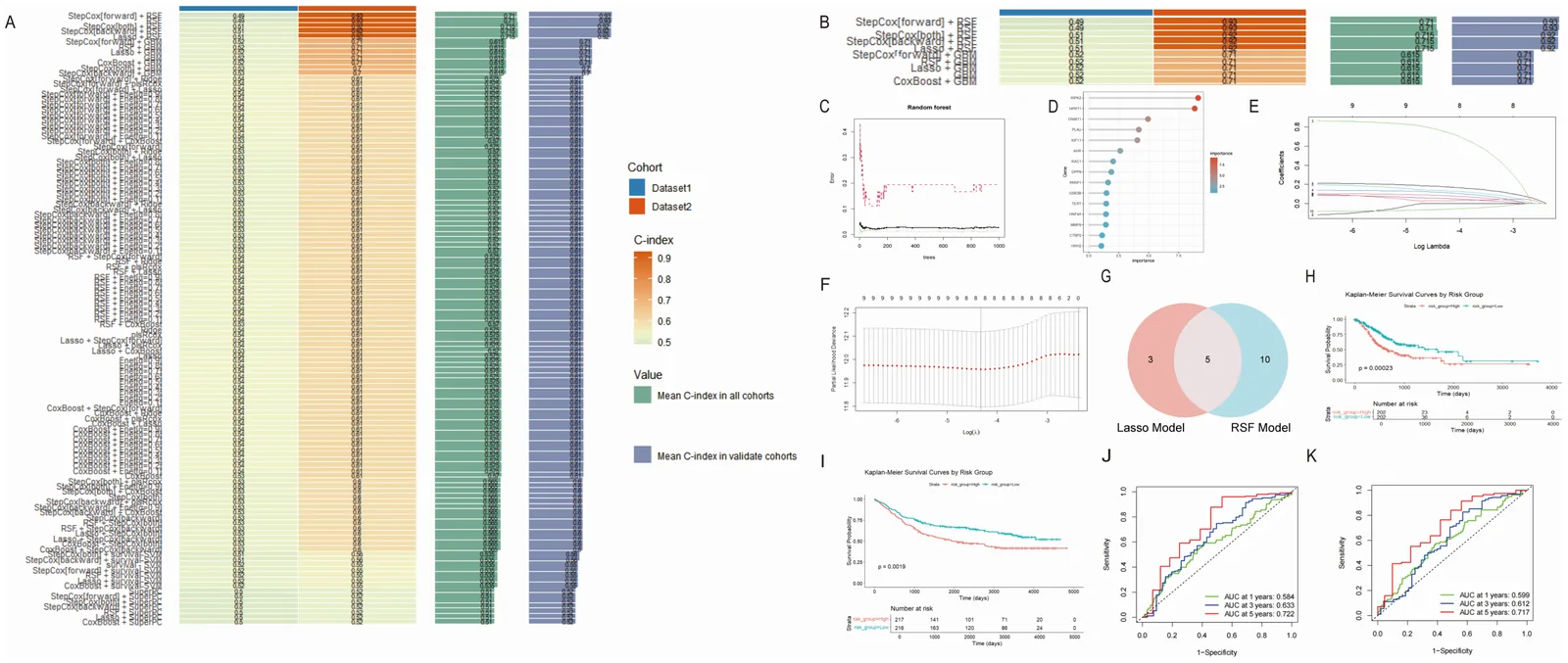
Development and external validation of the APRGSig prognostic model. **(A)** Post-selection comparison of Harrell C-indices for 117 candidate survival-modeling pipelines across TCGA-STAD (Dataset1) and GSE84437 (Dataset2). Pipeline development and selection were performed exclusively within TCGA-STAD using nested LOOCV; cohort-specific and summary C-indices were calculated subsequently for descriptive cross-cohort comparison. Green and blue columns indicate the mean C-index across displayed cohorts and the external-validation C-index, respectively. **(B)** Ten highest-performing pipelines in the post-selection cross-cohort comparison. **(C)** Random survival forest (RSF) error across tree numbers. **(D)** RSF variable-importance ranking, highlighting the 15 prioritized genes. **(E)** LASSO-Cox coefficient trajectories. **(F)** Ten-fold cross-validation for selection of the LASSO penalty parameter (λmin). **(G)** Intersection of RSF- and LASSO-Cox-prioritized genes, yielding the five-gene APRGSig comprising DNMT1, GSK3B, HNF4A, MMP9, and TERT. H–I. Kaplan–Meier overall-survival curves in TCGA-STAD **(H)** and GSE84437 **(I)** using the fixed TCGA-STAD median cutoff. **(J, K)** Time-dependent ROC curves for 1-, 3-, and 5-year overall survival in TCGA-STAD **(J)** and GSE84437 **(K)**.

The 63 prioritized candidates were evaluated across 117 prespecified survival-modeling pipelines, with model development and selection confined to TCGA-STAD. Nested LOOCV ranked pipelines by aggregated out-of-fold Harrell C-index, and the selected RSF–LASSO workflow achieved a pipeline-selection C-index of 0.715. After model selection and locking, cohort-specific performance profiles of the candidate pipelines were generated for descriptive cross-cohort comparison (**Figures 3A, B**), preserving the TCGA-STAD-only selection framework. Within the selected workflow, RSF retained 15 genes by variable importance, whereas LASSO-Cox retained eight genes at the cross-validated λmin; five genes were retained by both procedures—DNMT1, GSK3B, HNF4A, MMP9, and TERT—and constituted APRGSig (**Figures 3C–G**). Pollutant provenance linked DNMT1 to all seven pollutant-derived lists, GSK3B to benzene, HNF4A and MMP9 to SO_2_, and TERT to toluene and CO (**Supplementary Table 2B**).

APRGSig achieved an apparent Harrell C-index of 0.741 (95% CI, 0.712–0.769), a nested out-of-fold C-index of 0.713 (95% CI, 0.679–0.748), and an optimism-corrected C-index of 0.720 (95% CI, 0.686–0.754) in TCGA-STAD. GSE84437 comprised 433 eligible patients with 209 deaths and a median follow-up of 54.4 months (IQR, 27.2–86.9 months); Schoenfeld-residual testing supported the proportional-hazards assumption (P = 0.360). Locked external evaluation yielded a C-index of 0.701 (95% CI, 0.671–0.732), indicating preserved cross-cohort discrimination. Integrated Brier scores were 0.188 for nested TCGA-STAD predictions and 0.194 for GSE84437, with prediction-error curves favoring APRGSig over the Kaplan–Meier reference in both cohorts (**Supplementary Table 5**; **Supplementary Figure 1**). Using the fixed TCGA-STAD median cutoff, APRGSig-high patients had shorter overall survival in both cohorts (log-rank P < 0.05; **Figures 3H, I**). The 1-, 3-, and 5-year AUCs were 0.584, 0.633, and 0.722 in TCGA-STAD and 0.599, 0.612, and 0.717 in GSE84437 (**Figures 3J, K**).

### 3.7 Internal and external validation of the GC-ANN tissue classifier

**Figure 4:**
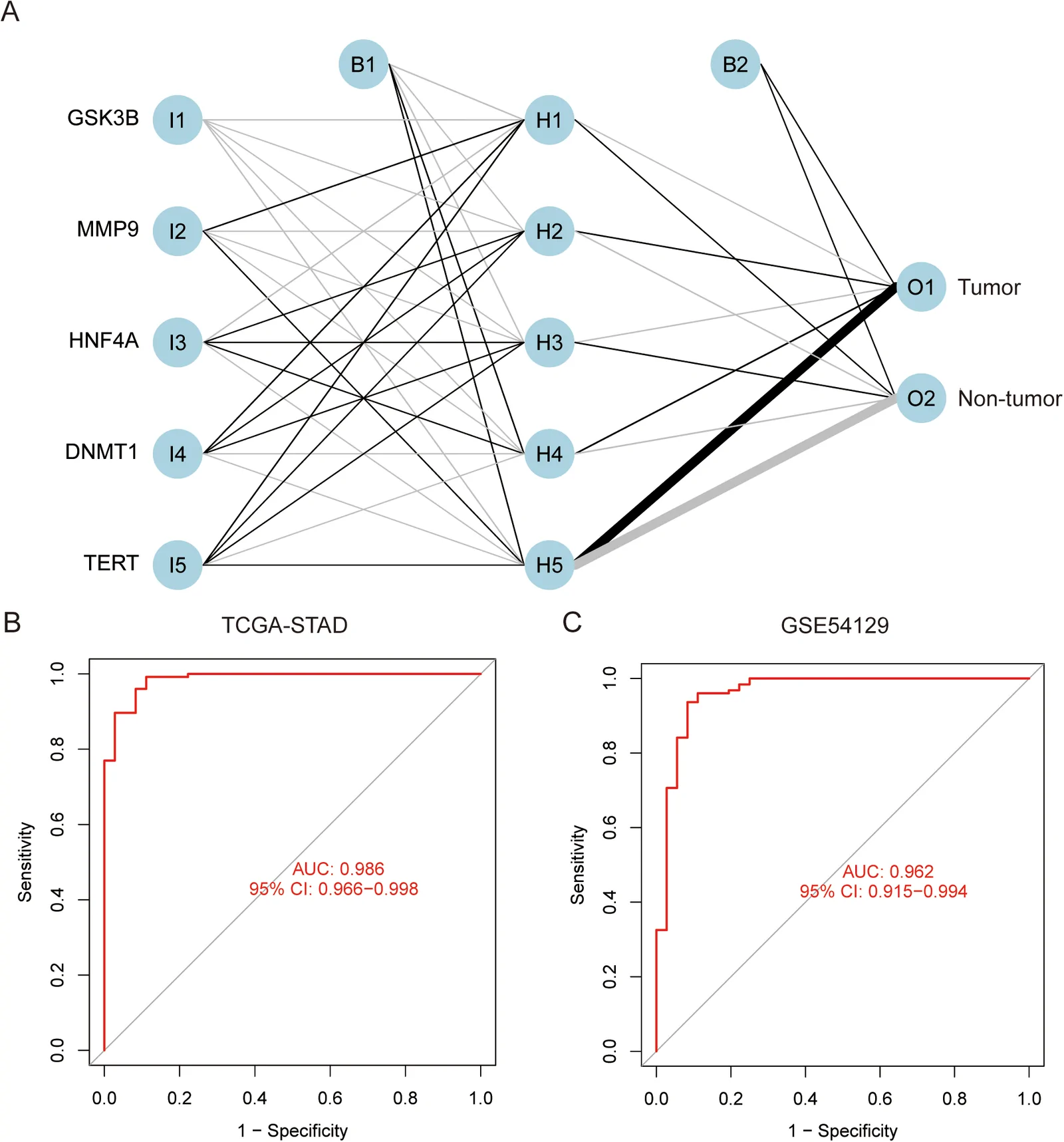
**(A)** Architecture of the multilayer perceptron comprising five input nodes corresponding to DNMT1, TERT, GSK3B, MMP9, and HNF4A, one hidden layer containing five neurons, and a two-node output layer representing gastric cancer and non-tumor gastric tissue. **(B)** Apparent receiver operating characteristic curve obtained after fitting and evaluating the ANN using the complete TCGA-STAD development cohort. **(C)** Receiver operating characteristic curve from the locked external evaluation in GSE54129.

The GC-ANN integrated the prespecified five-gene panel of DNMT1, TERT, GSK3B, MMP9, and HNF4A through a multilayer perceptron comprising five input nodes, one hidden layer with five neurons, and two output nodes representing gastric cancer and non-tumor gastric tissue (**Figure 4A**). In TCGA-STAD, the model achieved an apparent AUC of 0.986 (95% CI, 0.966–0.998; **Figure 4B**). Repeated nested cross-validation confirmed stable internal discrimination, with an out-of-fold AUC of 0.920 (95% CI, 0.870–0.962), while bootstrap optimism correction yielded an AUC of 0.921. At the prespecified threshold of 0.825, sensitivity, specificity, balanced accuracy, Matthews correlation coefficient, and Brier score were 0.912, 0.781, 0.847, 0.534, and 0.045, respectively (**Supplementary Table 6A**). After model locking, external evaluation in GSE54129 yielded an AUC of 0.962 (95% CI, 0.915–0.994; **Figure 4C**), with sensitivity, specificity, balanced accuracy, Matthews correlation coefficient, and Brier score of 0.919, 0.762, 0.840, 0.636, and 0.072, respectively. The calibration slope was 0.928 and intercept −0.498 (**Supplementary Table 6A**), supporting preserved probability ranking across cohorts.

Under the same validation framework, GC-ANN consistently outperformed the evaluated logistic, LASSO-logistic, random-forest, and single-gene models. Random forest, the strongest multivariable comparator, achieved AUCs of 0.873 internally and 0.889 externally; GC-ANN improved discrimination by ΔAUCs of 0.047 (95% CI, 0.022–0.079; P = 0.0010) and 0.073 (95% CI, 0.029–0.127; P = 0.0032), respectively. DNMT1 was the strongest single-gene classifier, with AUCs of 0.777 and 0.744, whereas the corresponding ANN–DNMT1 ΔAUCs were 0.143 and 0.218 (both P = 0.0002). GC-ANN also achieved the highest balanced accuracy and Matthews correlation coefficient and the lowest Brier score among the evaluated models (**Supplementary Table 6B**). Collectively, these results support the added discriminatory value and cross-cohort reproducibility of nonlinear integration of the five-gene panel for gastric tumor-versus-non-tumor tissue classification.

### 3.8 SHAP characterization of GC-ANN predictive contributions

**Figure 5:**
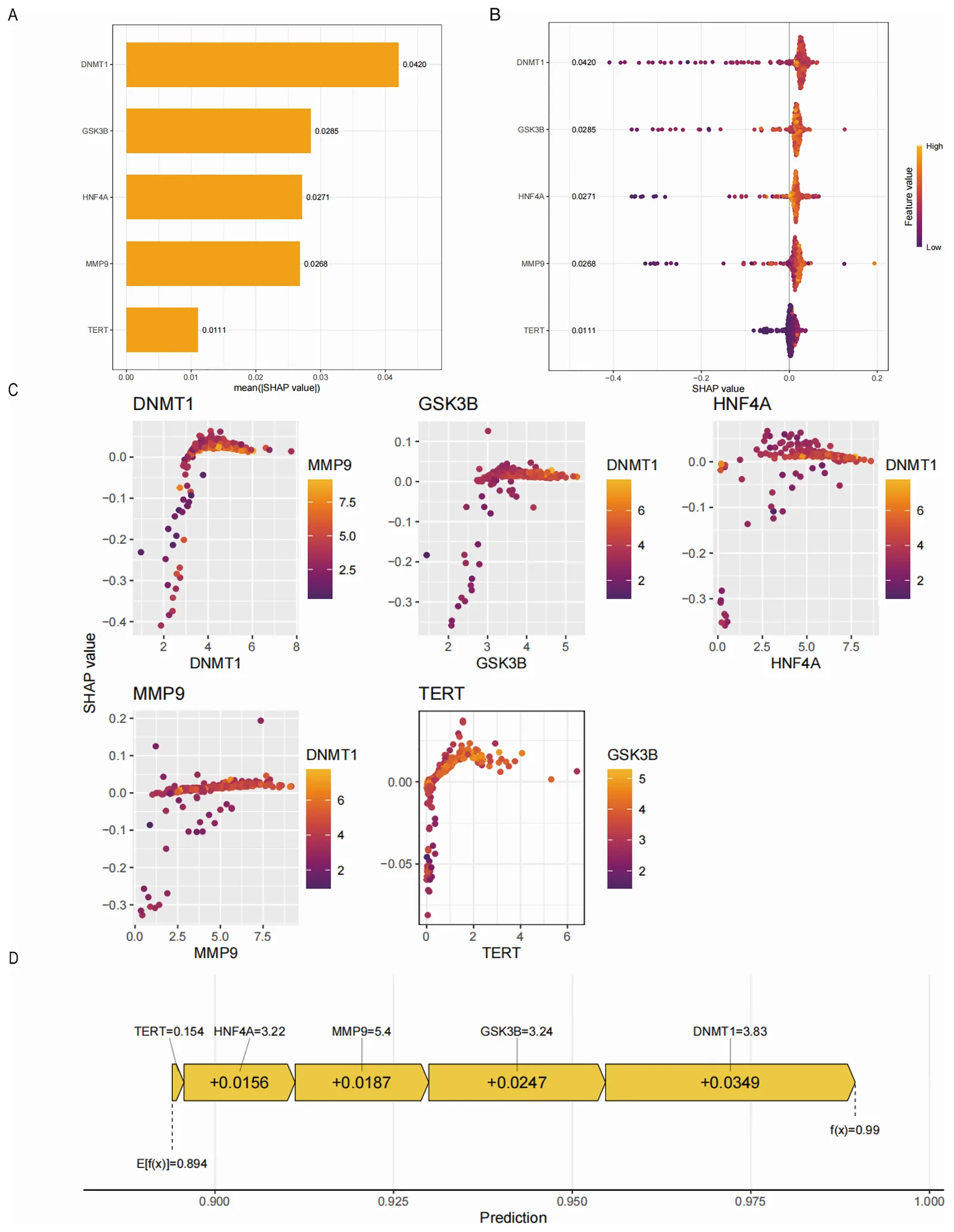
**(A)** Mean absolute SHAP values ranking the five input genes according to their overall contribution to GC-ANN predictions. **(B)** SHAP summary plot showing the distribution and direction of gene-specific predictive contributions across samples; color represents normalized gene expression. **(C)** SHAP dependence plots showing the relationships between gene-expression values and their contributions to the predicted probability of gastric cancer. **(D)** Representative force plot illustrating how the five gene-specific contributions combine to shift an individual prediction from the model reference value toward the predicted probability of gastric cancer.

SHAP analysis resolved the relative contributions of the five input genes to GC-ANN predictions on the predicted-probability scale. DNMT1 showed the largest mean absolute SHAP contribution, followed by GSK3B, HNF4A, MMP9, and TERT (**Figure 5A**). Summary and dependence plots showed that higher expression of DNMT1, GSK3B, HNF4A, and MMP9 generally contributed positively to the predicted probability of gastric cancer, whereas TERT displayed a weaker and more context-dependent contribution across its expression range (**Figures 5B, C**). In the representative tumor sample shown in **Figure 5D**, the five gene-specific contributions collectively shifted the model output from its reference value toward a higher predicted probability of gastric cancer, with DNMT1 and GSK3B providing the largest positive contributions in this example. Together, these patterns delineate how the fitted GC-ANN distributes predictive information across its five input features.

### 3.9 Transcript expression and survival associations of APRGSig genes

**Figure 6:**
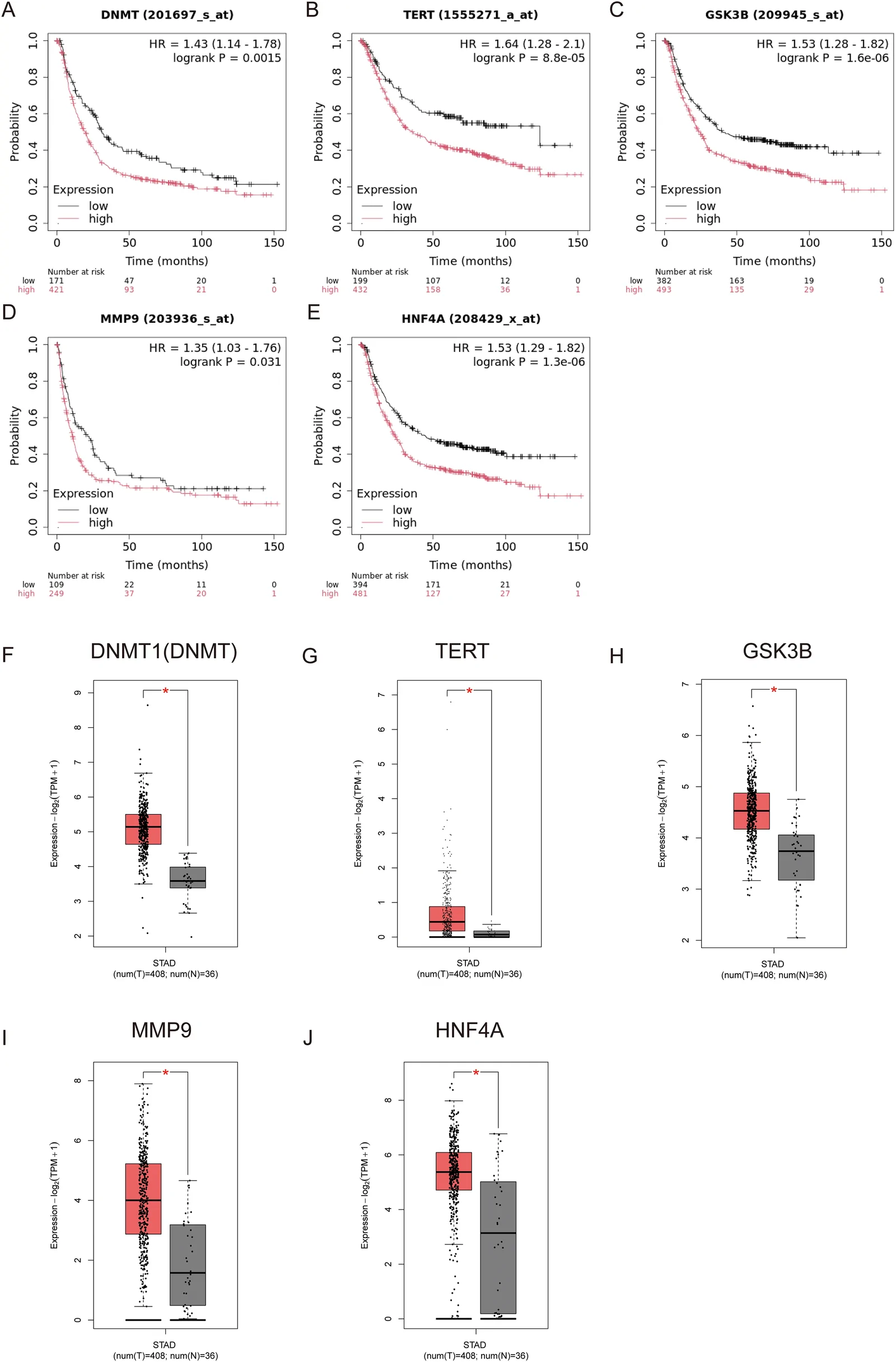
Expression patterns and overall-survival associations of APRGSig genes in gastric cancer. **(A–E)** Kaplan–Meier overall-survival curves for DNMT1, TERT, GSK3B, MMP9, and HNF4A. High- and low-expression groups were defined using gene-specific cutoffs selected by the Kaplan–Meier Plotter “auto select best cutoff” procedure. HRs and 95% CIs were estimated using univariate Cox proportional-hazards models, and survival distributions were compared using the log-rank test. **(F–J)** GEPIA comparisons of mRNA expression between TCGA-STAD gastric tumor samples (n = 408) and adjacent non-tumor gastric tissues (n = 36); GTEx normal tissues were not included.

Using gene-specific cutoffs selected by the Kaplan–Meier Plotter auto-selection procedure, high expression of DNMT1 (HR = 1.43), TERT (HR = 1.64), GSK3B (HR = 1.53), MMP9 (HR = 1.35), and HNF4A (HR = 1.53) was associated with shorter overall survival than the corresponding low-expression groups (all log-rank P < 0.05; **Figures 6A–E**). GEPIA analysis further showed higher mRNA expression of all five APRGSig genes in TCGA-STAD gastric tumors than in TCGA-STAD adjacent non-tumor gastric tissues (all P < 0.05; **Figures 6F–J**). ShinyTHOR profiling further showed heterogeneous baseline expression of the five APRGSig genes across established stomach carcinoma cell lines (**Supplementary Figure 2**).

### 3.10 Development and assessment of the prognostic nomogram

**Figure 7:**
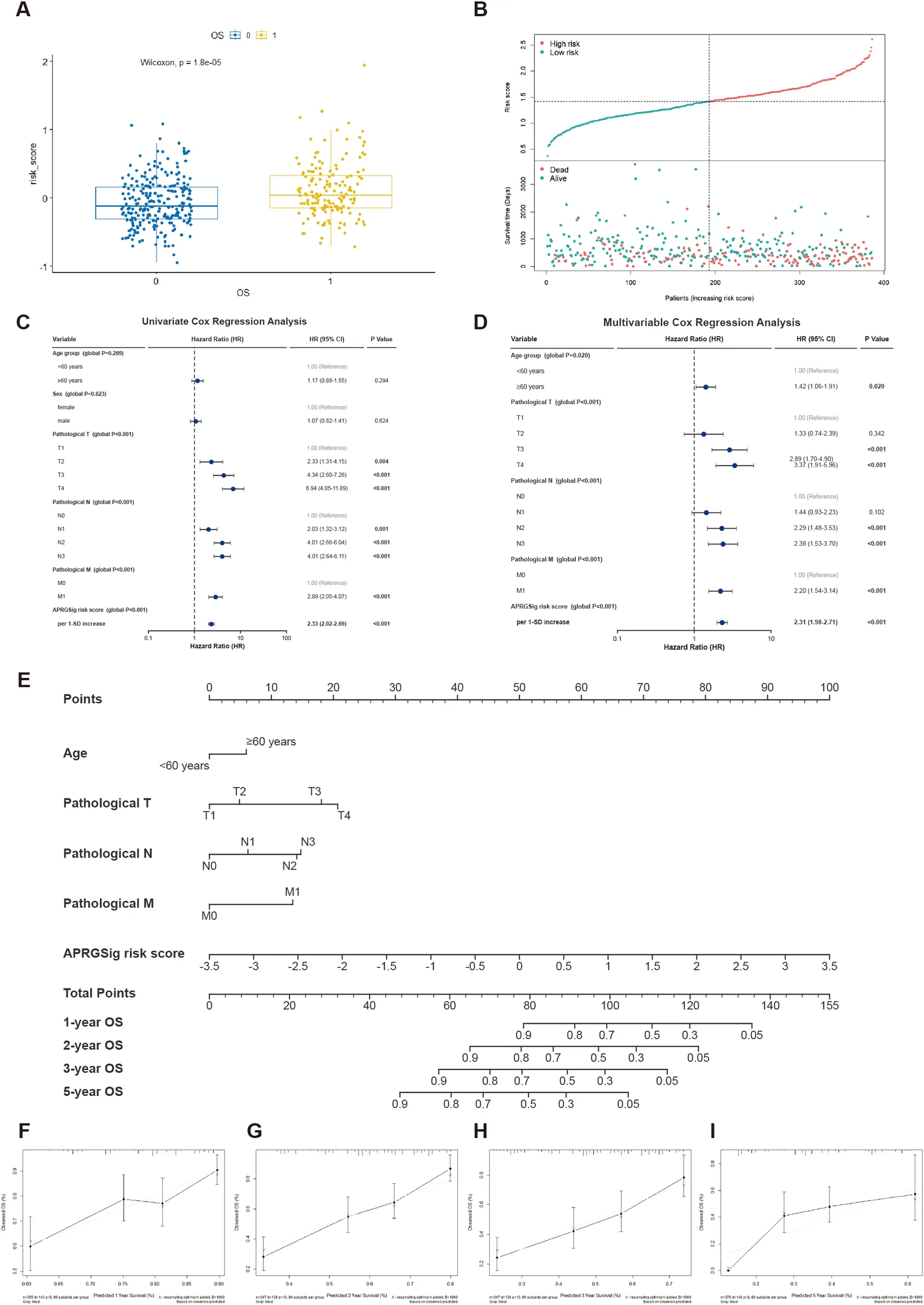
**(A)** Distribution of APRGSig risk scores according to survival status. **(B)** Distribution of APRGSig risk scores, survival time, and event status across patients. **(C)** Forest plot of univariate Cox regression analyses. **(D)** Forest plot of multivariable Cox regression analysis. **(E)** Prognostic nomogram for estimating 1-, 2-, 3-, and 5-year overall survival. **(F–I)**. Calibration curves for predicted versus observed 1-, 2-, 3-, and 5-year overall survival, respectively.

Patients with an observed death event had higher APRGSig risk scores than those without an event (Wilcoxon P = 1.8×10⁻^5^; **Figure 7A**), and their risk-score, survival-time, and event distributions are shown in **Figure 7B**. In univariate Cox analysis, pathological T, N, and M stages and APRGSig risk score were associated with overall survival, whereas age group and sex were not (**Figure 7C**). APRGSig showed a strong association with shorter survival (HR = 2.33 per 1-SD increase; 95% CI, 2.02–2.69; P<0.001). In multivariable analysis, APRGSig remained independently associated with shorter survival (HR = 2.31; 95% CI, 1.98–2.71; P<0.001), while age ≥60 years, T3/T4, N2/N3, and M1 also retained significant associations (**Figure 7D**). These variables formed the prognostic nomogram (**Figure 7E**), and calibration curves showed overall agreement between predicted and observed 1-, 2-, 3-, and 5-year survival probabilities (**Figures 7F–I**).

### 3.11 Single-cell analysis

**Figure 8:**
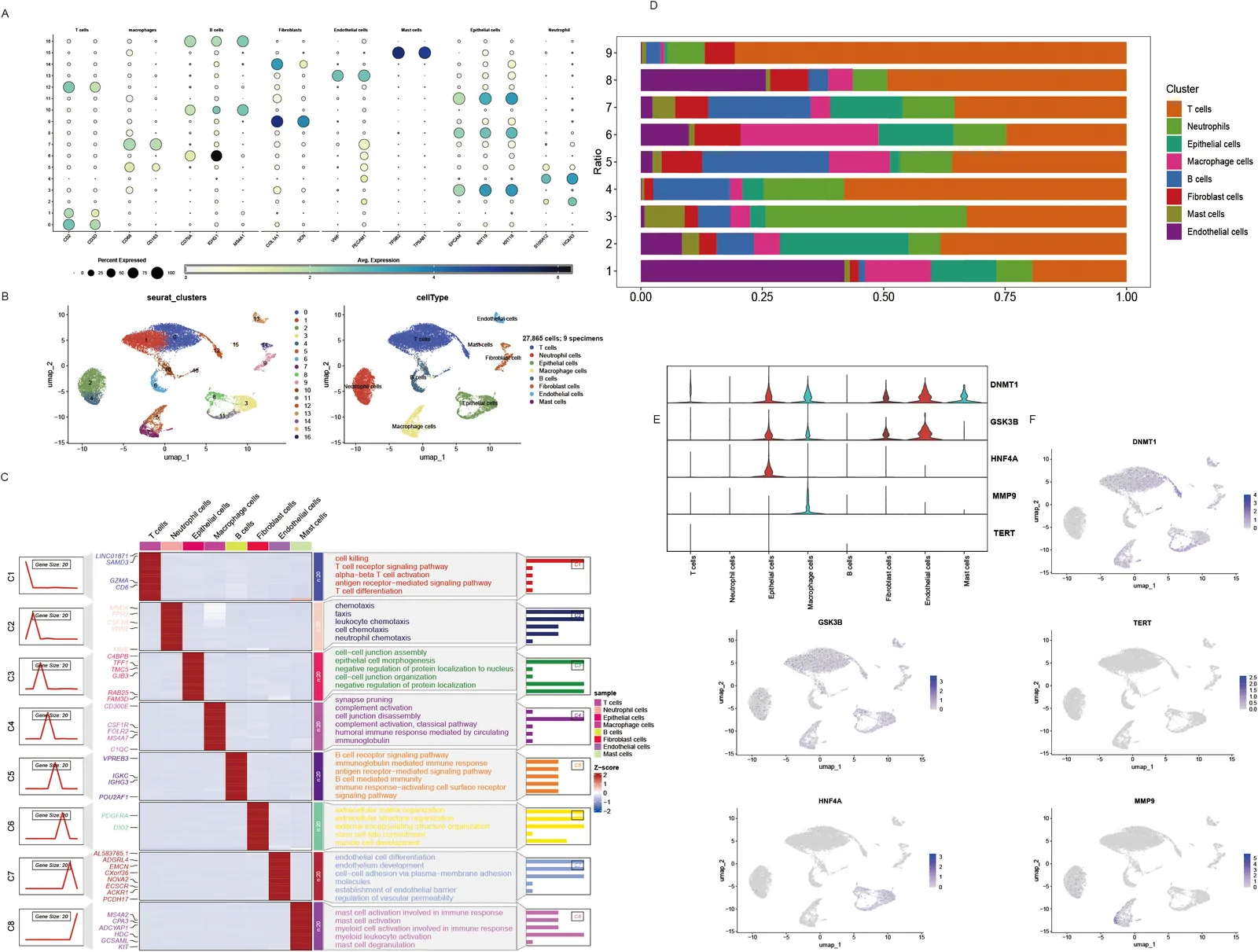
**(A)** Marker genes employed for cell‐type annotation. **(B)** UMAP projection delineating 17 discrete cell clusters; eight of these clusters were annotated based on canonical marker gene expression. **(C)** The heatmap illustrates the top 10 marker genes ranked in each cell cluster. **(D)** Bar graph illustrating the relative abundance of major cell populations in each sample. **(E, F)** Violin plots showing the transcript-expression distributions of the five APRGSig genes across annotated cell types.

Harmony integration produced concordant cellular distributions across samples, indicating minimal batch effects and supporting downstream analysis. At a resolution of 0.4, 27,865 scRNA-seq profiles from nine specimens, comprising primary gastric tumors and lymph node, hepatic, and ovarian metastases, resolved into 17 clusters. Leveraging canonical marker genes, we annotated eight major cell lineages: T cells, B cells, epithelial cells, macrophages, neutrophils, mast cells, fibroblasts and endothelial cells (**Figures 8B, D**). Distinct expression patterns of lineage‐specific markers corroborated the accuracy of our annotations (**Figure 8A**). We then assessed the distribution of five hub genes (DNMT1, GSK3B, HNF4A, MMP9 and TERT) across these defined cell types (**Figures 8E, F**). HNF4A was sharply enriched within epithelial cells. MMP9 expression was largely restricted to the macrophage lineage and was negligible in other compartments. DNMT1 and GSK3B exhibited broad, multi-lineage expression spanning epithelial, endothelial, fibroblast, and macrophage populations, with DNMT1 additionally detectable in mast cells. By contrast, TERT showed uniformly low abundance and was predominantly confined to epithelial cells. Collectively, these data delineate the cell type–specific expression landscape of our five hub genes within the gastric tumor microenvironment.

Cluster-specific differentially expressed genes further supported the cell annotations (**Figure 8C**). GO analysis showed immune-response enrichment in T cells, macrophages, B cells, and mast cells, whereas epithelial-cell genes were enriched in cell–cell junction processes.

### 3.12 Representative compartment-level localization of APRGSig-encoded proteins in human gastric cancer

**Figure 9:**
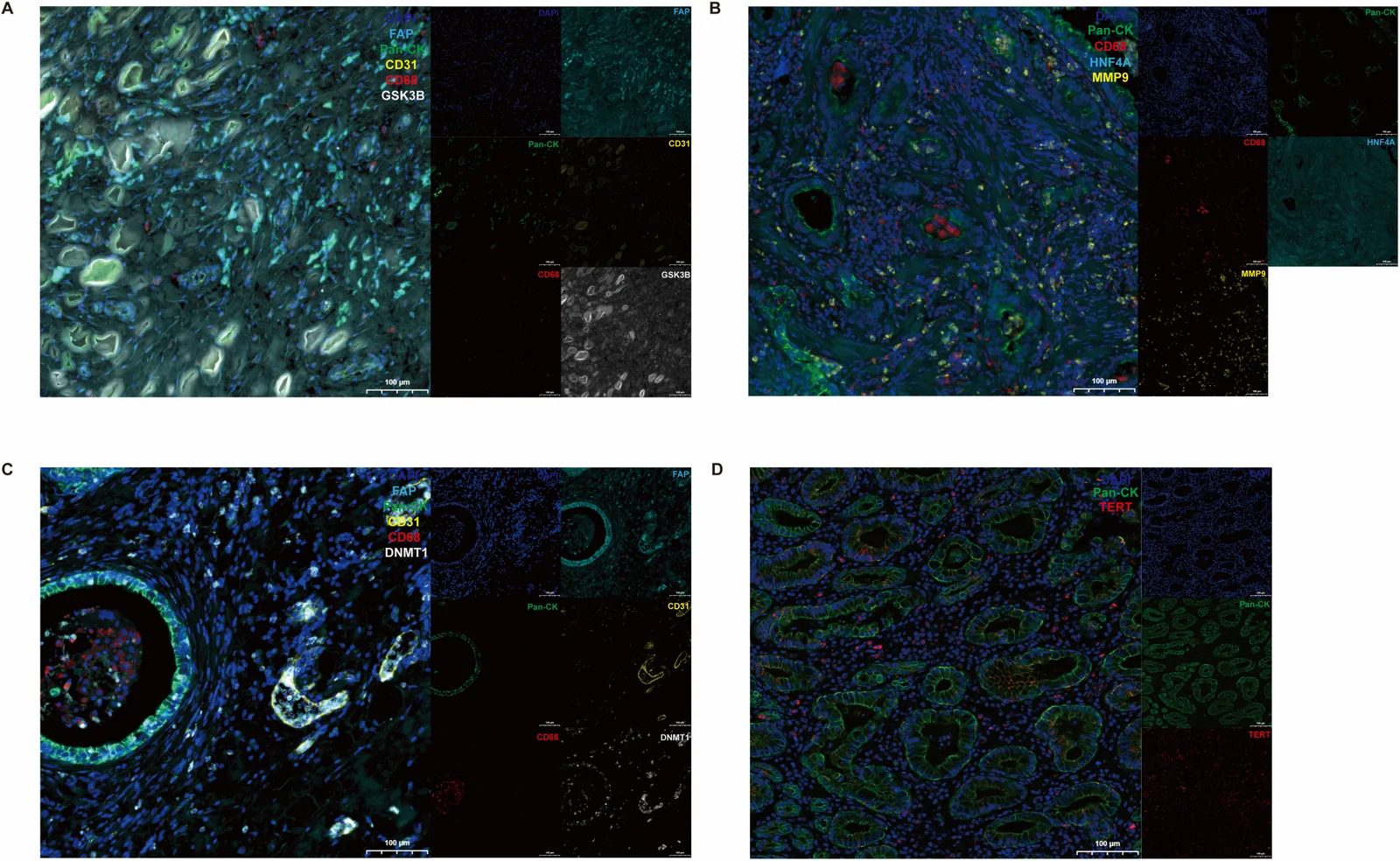
Representative compartment-level localization of APRGSig-encoded proteins in human gastric cancer tissues. Representative regions from four patients are shown. **(A)** Multiplex immunofluorescence showing GSK3B signals (white) in CD31^+^ endothelial structures (yellow), Pan-CK^+^ epithelial tumor regions (green), CD68^+^ macrophage-associated regions (red), and FAP^+^ stromal compartments (cyan). **(B)** HNF4A (cyan) and MMP9 (yellow) signals were visually observed within or adjacent to representative Pan-CK^+^ epithelial regions (green) and CD68^+^ macrophage-associated regions (red). **(C)** DNMT1 signals (white) were detectable across CD31^+^ endothelial, Pan-CK^+^ epithelial, CD68^+^ macrophage-associated, and FAP^+^ stromal compartments. **(D)** TERT signals (red) appeared mainly as discrete punctate staining within or adjacent to Pan-CK^+^ epithelial tumor regions (green). Nuclei were counterstained with DAPI (blue). Merged and corresponding single-channel images are shown. Pan-CK, CD68, CD31, and FAP were used to identify epithelial, macrophage-associated, endothelial, and stromal compartments, respectively. Scale bars, 100 μm in all panels.

Across representative tissue regions from four patients, multiplex immunofluorescence provided orthogonal localization evidence for DNMT1, TERT, GSK3B, MMP9, and HNF4A in human gastric cancer tissues. GSK3B and DNMT1 signals showed relatively broad tissue distributions, with prominent staining in Pan-CK^+^ epithelial tumor nests and detectable signals in CD68^+^ macrophage-associated regions, CD31^+^ vascular structures, and adjacent FAP^+^ stromal compartments (**Figures 9A, C**). MMP9 and HNF4A signals were visually observed within or adjacent to representative Pan-CK^+^ epithelial and CD68^+^ macrophage-associated regions (**Figure 9B**). TERT staining was comparatively sparse and appeared mainly as discrete punctate signals within or adjacent to Pan-CK^+^ epithelial tumor glands (**Figure 9D**). Isotype-control sections showed no specific compartment-associated staining. Together, these *in situ* localization patterns were concordant with the cell type-resolved transcriptomic profiles and supported compartment-level localization of APRGSig-encoded proteins.

### 3.13 Characterization of APRGSig-related genes in gastric cancer scRNA-seq data

**Figure 10:**
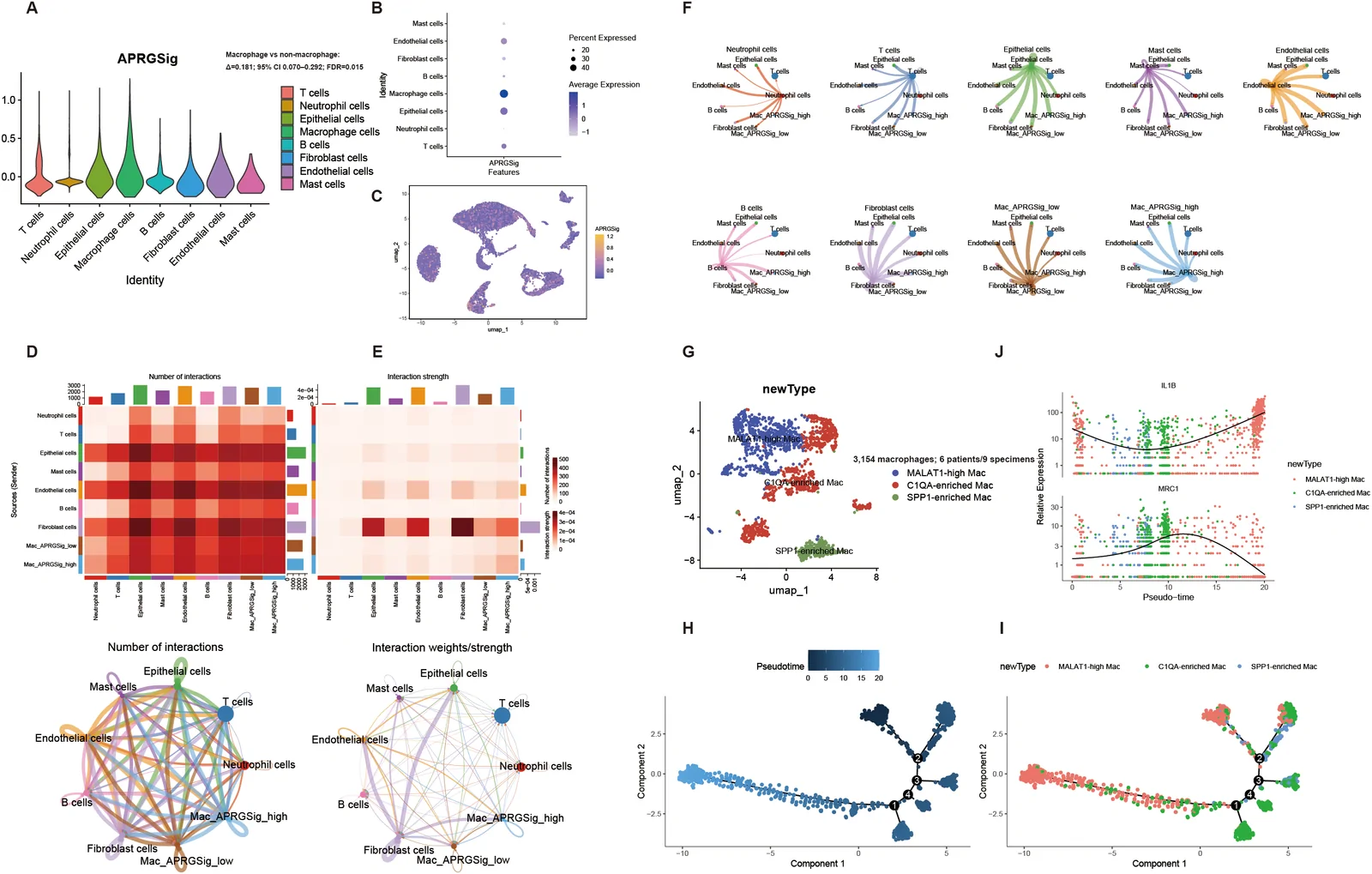
**(A)** The violin plot comparing APRGSig scores across the eight cell types was generated. **(B)** Dot plot illustrating the expression of APRGSig in different cell types. **(C)** UMAP projection of the APRGSig scores across individual cells in the gastric tumor dataset. **(D)** Network diagram and accompanying heatmap illustrating the frequency of intercellular communication events among nine defined cell types. **(E)** Network diagram and accompanying heatmap depicting the strength of cell-cell interaction signals among the same nine defined cell types. **(F)** Quantitative summary of both the number and the average intensity of communication events between each pair of defined cell types. **(G)** UMAP plot showing the distribution of macrophage subtypes, including MALAT1-high macrophage (blue), C1QA-enriched macrophage (red) and SPP1-enriched macrophage (green), identified through single-cell analysis. **(H)** Monocle2-inferred pseudotemporal ordering of macrophages colored by pseudotime. **(I)** Distribution of MALAT1-high, C1QA-enriched, and SPP1-enriched macrophages along the inferred pseudotemporal continuum. **(J)** Expression trends of IL1B and MRC1 across pseudotime, with cells colored by macrophage state and curves indicating fitted expression trajectories.

The five prognostic genes, DNMT1, GSK3B, HNF4A, MMP9, and TERT, were scored as APRGSig across cell types using Seurat AddModuleScore (**Figures 10A–C**). Donor-aware analysis showed higher APRGSig scores in macrophages than in non-macrophage cells, with patient-level differences of 0.181 by AddModuleScore (95% CI, 0.070–0.292; FDR = 0.015) and 0.121 by UCell (95% CI, 0.073–0.169; FDR = 0.008; **Supplementary Table 7A**). Gene-level decomposition identified MMP9 as the largest positive contributor (36.6%), followed by DNMT1 (23.4%) and GSK3B (19.8%; **Supplementary Table 7B**). Excluding MMP9 attenuated but did not eliminate the macrophage-associated contrast (difference=0.070; 95% CI, 0.004–0.136; FDR = 0.042; **Supplementary Table 7A**). The observed contrast also exceeded 99.96% of 5,000 expression-matched random signatures (empirical P = 0.0006; **Supplementary Table 7C**), supporting a reproducible multigene signal with MMP9 as its strongest, but not sole, contributor.

### 3.14 Cell–cell communication inference

For CellChat analysis, macrophages in each specimen were divided at the specimen-specific median APRGSig score into APRGSig-high and APRGSig-low groups. Together with seven non-macrophage lineages, this yielded nine groups (**Figures 10D–F**). APRGSig-high macrophages had greater inferred incoming and outgoing communication strengths, particularly with fibroblasts, endothelial cells, and epithelial cells (**Supplementary Figure 3A**). Incoming signals involved COLLAGEN, FN1, APP, MHC-I, and MHC-II pathways, whereas outgoing signals included SPP1, CXCL, MHC-I, MHC-II, and GALECTIN pathways (**Supplementary Figure 3B**). Representative predicted interactions comprised COL1A1/2–CD44, COL1A1/2–integrin, APP–CD74, SPP1–integrin, and CXCL8–ACKR1 (**Supplementary Figure 3C**). Across pooled computational predictions, these inferences linked APRGSig-high macrophages to stromal, vascular, and epithelial compartments.

### 3.15 Construction of the macrophage atlas and pseudotime analysis

The 3,154 macrophages were reclustered into eight transcriptional clusters (**Supplementary Figure 4A**) and consolidated, using differential expression and canonical myeloid markers, into three computationally defined states: MALAT1-high, C1QA-enriched, and SPP1-enriched macrophages (**Figure 10G**; **Supplementary Figure 4B**). These transcriptional states were defined independently of the APRGSig-high/low score groups used for CellChat. MALAT1-high macrophages comprised 1,229 cells and were detected in all nine specimens, including all three primary tumors and all six metastatic specimens (**Supplementary Table 7D**). Within the three primary tumors, they accounted for 31.2–43.2% of macrophages, supporting consistent detection in the primary-tumor context. Patient-level sequencing depth, detected-feature number, mitochondrial-transcript percentage, doublet proportion, and ambient-RNA score were comparable across states after FDR correction (**Supplementary Table 7E**). Re-clustering after excluding MALAT1 from highly variable feature selection retained 88.7% of the original MALAT1-high cells (Jaccard=0.831; adjusted Rand index=0.870), together with MALAT1-independent inflammatory markers including IL1B, FCN1, CXCL8, CCL3, CCL4, NFKBIA, and TNFAIP3 (**Supplementary Table 8**).

**Figure 11:**
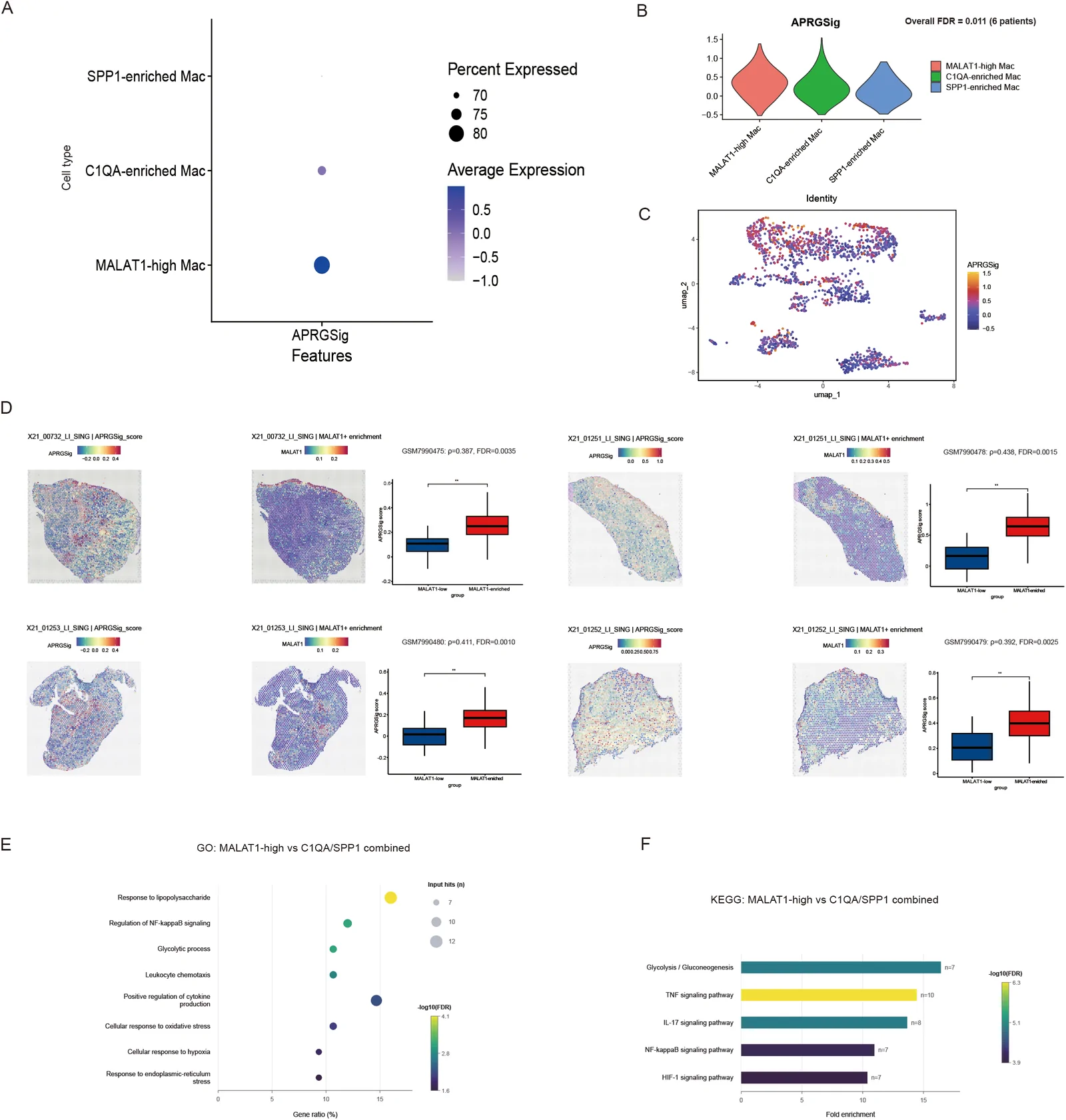
APRGSig module scoring characterizes an APRGSig-enriched MALAT1-high macrophage state and its spatial and functional associations in gastric cancer. **(A–C)** Distribution of APRGSig_Score across annotated macrophage subsets shown as a dot plot **(A)**, violin plot **(B)**, and UMAP projection **(C)**. **(D)** Spatial distributions of APRGSig scores and cell2location-inferred MALAT1-high macrophage abundance across four gastric cancer Visium sections. **FDR<0.01. **(E)** GO biological-process enrichment of the 75 genes upregulated in MALAT1-high macrophages versus the combined C1QA-enriched/SPP1-enriched group. Dot position, size, and color indicate gene ratio, gene count, and −log10(FDR), respectively. **(F)** Selected KEGG pathways enriched among the same genes. Bar length, color, and n indicate fold enrichment, −log10(FDR), and gene count, respectively.

Donor-aware analysis showed the highest APRGSig scores in MALAT1-high macrophages, followed by C1QA-enriched and SPP1-enriched states, with patient-level AddModuleScore means of 0.498, 0.414, and 0.361, respectively (overall FDR = 0.011; **Figures 11A–C**; **Supplementary Table 7F**). This ordering was preserved after MMP9 exclusion (0.329, 0.279, and 0.247; overall FDR = 0.028) and with UCell scoring (0.708, 0.667, and 0.618; overall FDR = 0.014). The MALAT1-high–versus–other-state contrast exceeded 99.92% of 5,000 expression-matched random signatures (empirical P = 0.0010; **Supplementary Table 7C**).

Donor-blocked pseudo-bulk analysis identified 75 upregulated and 65 downregulated genes in MALAT1-high macrophages, with increased IL1B, CXCL8, CCL3, CCL4, NFKBIA, TNFAIP3, PLAUR, SOD2, FCN1, and NAMPT and reduced MRC1 and selected homeostatic markers (**Supplementary Table 7G**). Enrichment highlighted cytokine production, leukocyte chemotaxis, oxidative stress, hypoxia, glycolysis, TNF, NF-κB, IL-17, and HIF-1 signaling (**Figures 11E, F**; **Supplementary Table 7H**). Exploratory Monocle analysis positioned SPP1-enriched cells earlier, C1QA-enriched cells in intermediate regions, and MALAT1-high cells later along a branched pseudotemporal continuum (**Figures 10H, I**), accompanied by increasing IL1B and decreasing MRC1 expression (**Figure 10J**). Together, these results characterize MALAT1-high macrophages as a reproducible inflammatory/stress-associated transcriptional state with enriched APRGSig activity.

### 3.16 Spatial transcriptomic analysis

Four gastric cancer Visium sections were analyzed independently to characterize the spatial relationship between APRGSig and inferred MALAT1-high macrophage abundance. Regions with elevated APRGSig scores frequently overlapped or bordered regions with greater inferred MALAT1-high abundance (**Figure 11D**). APRGSig showed positive spatial autocorrelation across GSM7990480, GSM7990479, GSM7990478, and GSM7990475 (Moran’s I = 0.317, 0.291, 0.307, and 0.314, respectively; FDR = 0.0010–0.0020), as did inferred MALAT1-high abundance (Moran’s I = 0.277, 0.253, 0.280, and 0.264; FDR = 0.0012–0.0030). Spot-level concordance was consistently positive (Spearman ρ=0.411, 0.392, 0.438, and 0.387; FDR = 0.0010–0.0035), together with positive neighborhood-aware cross-association (bivariate Moran’s I = 0.220, 0.218, 0.227, and 0.213; permutation FDR = 0.0015–0.0032; **Supplementary Table 9**). Collectively, these directionally consistent spatial statistics across four independent sections provide reproducible quantitative support for spatial concordance between APRGSig-enriched regions and inferred MALAT1-high macrophage abundance in gastric cancer.

### 3.17 Structural modeling of benzene and toluene with APRGSig-encoded proteins

**Figure 12:**
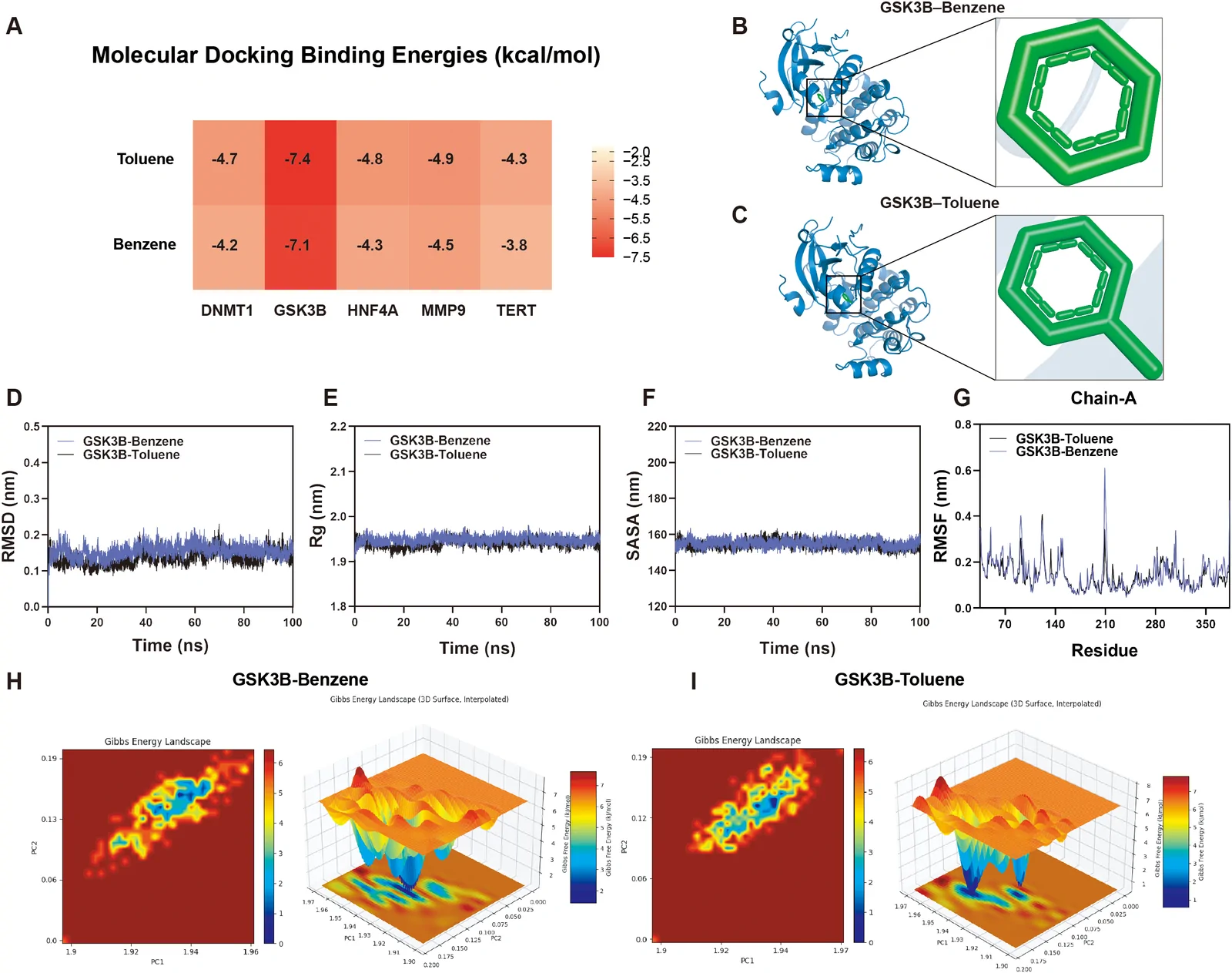
**(A)** Heatmap of AutoDock Vina docking scores (kcal/mol) for benzene and toluene across the five candidate proteins (DNMT1, GSK3B, HNF4A, MMP9, and TERT) and two air pollutants (benzene and toluene). **(B)** Visualization of molecular docking results for GSK3B and benzene. **(C)** Visualization of molecular docking results for GSK3B and toluene. **(D)** Time evolution of root mean square deviation (RMSD) for the protein–ligand complex. **(E)** Temporal variation of the radius of gyration (Rg) for the protein–ligand complex. **(F)** Time-dependent solvent-accessible surface area (SASA) of the protein–ligand complex. **(G)** Time-resolved profile of root mean square fluctuation (RMSF) for the protein–ligand complex. **(H)** Relative conformational free-energy landscape of the GSK3B–benzene model constructed using RMSD and radius of gyration as collective coordinates. **(I)** Relative conformational free-energy landscape of the GSK3B–toluene model constructed using RMSD and radius of gyration as collective coordinates. During the 100-ns simulations, both GSK3B models showed initial conformational adjustment followed by comparatively limited variation. RMSD stabilized during later trajectory intervals, while radius of gyration and SASA remained within narrow ranges **(D–F)**. RMSF remained low across most of GSK3B chain A, with increased mobility confined to selected flexible regions **(G)**. RMSD–radius-of-gyration landscapes further revealed dominant low-relative-free-energy basins within the sampled conformational spaces **(H, I)**. Collectively, the docking controls, comparative scoring, and trajectory characteristics support the numerical persistence and structural plausibility of the modeled GSK3B–benzene and GSK3B–toluene configurations, prioritizing them as testable structural hypotheses for subsequent mechanistic investigation.

Structural modeling of benzene and toluene across the five APRGSig-encoded proteins identified GSK3B as the most prominent candidate for focused trajectory analysis. Among the evaluated complexes, GSK3B–benzene and GSK3B–toluene showed comparatively favorable AutoDock Vina scores and plausible pocket poses (**Figures 12A–C**). Redocking of the co-crystallized ADP accurately reproduced its crystallographic pose, with a median symmetry-corrected heavy-atom RMSD of 0.261 Å across three runs (range, 0.246–0.284 Å), satisfying the prespecified RMSD ≤2.0 Å criterion. Median Vina scores were −9.1 kcal/mol for CHIR-99021, −8.6 kcal/mol for ADP, −3.4 kcal/mol for cyclohexane, −7.1 kcal/mol for benzene, and −7.4 kcal/mol for toluene (**Supplementary Table 10**).

### 3.18 Bibliometric analysis of GSK3B in gastric cancer

**Figure 13:**
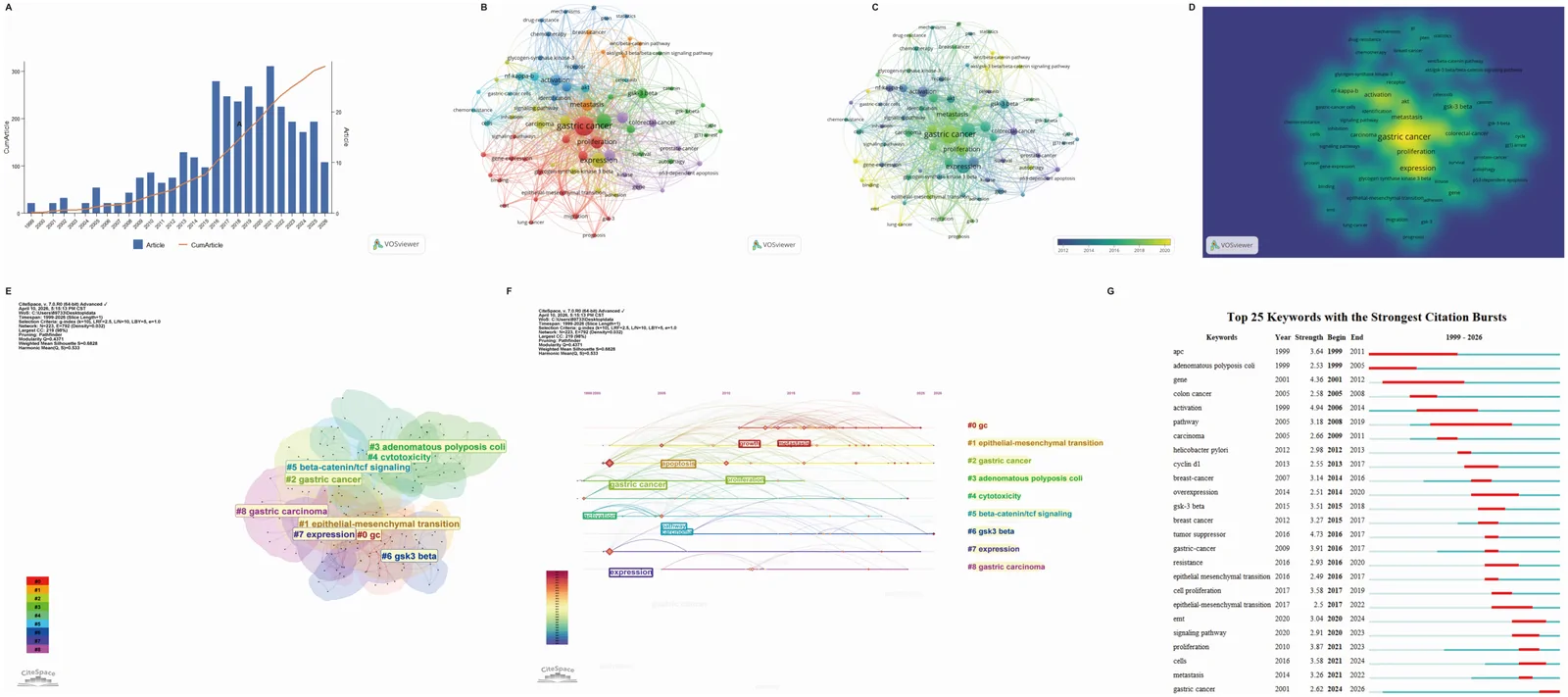
Bibliometric landscape of GSK3B research in gastric cancer. **(A)** Annual publication output and cumulative number of publications. **(B)** Keyword co-occurrence network showing major research clusters, generated by VOSviewer. **(C)** Temporal overlay visualization of keywords generated by VOSviewer, illustrating the evolution of research topics over time. **(D)** Density visualization of keywords generated by VOSviewer, highlighting high-frequency research themes. **(E)** Keyword cluster map generated by CiteSpace, showing the thematic structure of the field. **(F)** Timeline view of keyword clusters generated by CiteSpace, depicting the temporal progression of major research themes. **(G)** Top 25 keywords with the strongest citation bursts, indicating emerging fronts and periods of intensified research attention.

The bibliometric search identified 311 publications on GSK3B and gastric cancer, comprising 301 original articles and 10 reviews. Annual and cumulative output increased from 1999 to 2026, indicating sustained growth within a moderately sized literature (**Figure 13A**). Keyword co-occurrence linked gastric cancer and GSK3B to proliferation, apoptosis, progression, metastasis, invasion, β-catenin, Wnt signaling, and epithelial–mesenchymal transition (**Figure 13B**). Temporal mapping showed a shift from pathway activation, apoptosis, β-catenin, and Wnt-related signaling toward proliferation, invasion, metastasis, epithelial–mesenchymal transition, drug resistance, and treatment response (**Figure 13C**). Density mapping centered on gastric cancer, GSK3B expression, apoptosis, proliferation, metastasis, and invasion (**Figure 13D**). CiteSpace identified clusters for gastric cancer, epithelial–mesenchymal transition, adenomatous polyposis coli, cytotoxicity, β-catenin/TCF signaling, GSK3B, and gene expression, with a timeline progressing from disease- and expression-related work to pathway-, invasion-, metastasis-, and treatment-focused research (**Figures 13E, F**). “Activation” had the strongest historical citation burst, whereas “epithelial–mesenchymal transition,” “EMT,” “signaling pathway,” “proliferation,” and “metastasis” remained prominent more recently (**Figure 13G**). Together, these analyses delineated publication growth and thematic evolution.

### 3.19 Benzene and toluene alter viability, clonogenic capacity and motility-associated phenotypes in AGS cells

**Figure 14:**
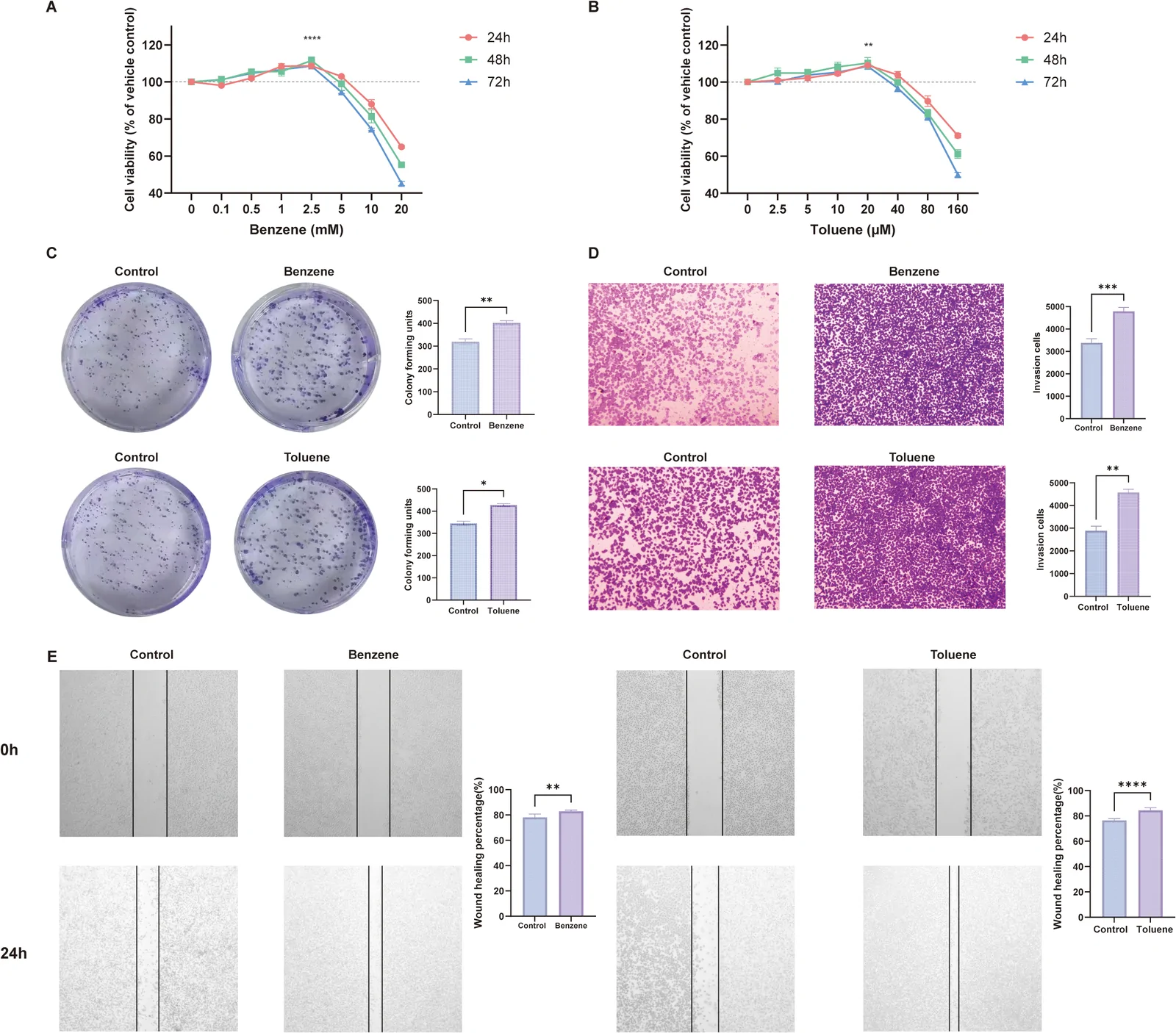
Phenotypic responses of AGS cells to benzene and toluene exposure. **(A, B)**, Concentration- and time-dependent effects of benzene (0–20 mM; A) and toluene (0–160 μM; B) on AGS-cell viability measured by CCK-8 assay at 24, 48, and 72 h. Viability was normalized to the corresponding vehicle control. Red circles, green squares, and blue triangles indicate 24-, 48-, and 72-h measurements, respectively. **(C)**, Representative colony-formation images and quantification after exposure to 2.5 mM benzene or 20 μM toluene for 8 days. **(D)**, Representative images and quantification of cells traversing Matrigel-coated Transwell inserts after 24 h of exposure. **(E)**, Representative wound-healing images at 0 and 24 h and quantification of wound closure. All quantitative data are presented as mean ± SD from three independent biological experiments (n = 3), each containing three technical replicate wells that were averaged within each experiment. CCK-8 data were analyzed separately for benzene and toluene using two-way ANOVA followed by Dunnett’s multiple-comparison test against the corresponding vehicle control within each time point; colony-formation, Transwell, and wound-healing data were analyzed using one-way ANOVA with Dunnett’s correction. *P < 0.05, **P < 0.01, ***P < 0.001, ****P < 0.0001.

We first characterized the concentration- and time-dependent effects of benzene and toluene on AGS cell viability. Both compounds exhibited non-linear viability profiles across the tested concentration ranges (**Figures 14A, B**). Viability was highest and most consistently maintained at 2.5 mM benzene and 20 μM toluene over the 24–72-h observation period, whereas higher concentrations produced progressively greater reductions in viability, particularly with prolonged exposure. Notably, 10–20 mM benzene and 80–160 μM toluene were associated with marked decreases in viability. Accordingly, 2.5 mM benzene and 20 μM toluene were selected as the working concentrations for subsequent AGS-cell phenotyping.

At these concentrations, both compounds enhanced the clonogenic capacity of AGS cells. Benzene significantly increased colony formation relative to the corresponding vehicle control (P < 0.01), and a similar increase was observed following toluene exposure (P < 0.05; **Figure 14C**). These findings extend the CCK-8 observations by demonstrating increased clonogenic capacity under the selected working conditions. Benzene and toluene also enhanced motility-associated phenotypes. In Matrigel-coated Transwell assays, significantly greater numbers of cells traversed the membrane following benzene (P < 0.001) or toluene (P < 0.01) exposure (**Figure 14D**). Consistently, both compounds increased wound closure over 24 h, with significant effects observed for benzene (P < 0.01) and toluene (P < 0.0001; **Figure 14E**). Collectively, the concentration–response, clonogenic, Transwell, and wound-healing assays demonstrate concordant phenotype-level responses to benzene and toluene in AGS cells, supporting a phenotype characterized by maintained viability, enhanced clonogenic capacity, increased Matrigel traversal, and accelerated wound closure under the tested conditions.

## 4 Discussion

Air pollution is one of the major environmental health threats faced by contemporary society, originating from a wide range of sources, including industrial emissions, vehicle exhaust, coal combustion, and biomass burning (22). These pollutants not only cause direct damage to the human respiratory and cardiovascular systems but are also closely associated with the development of various chronic diseases and cancers (23). Studies have demonstrated that air pollutants may influence physiological functions and promote disease progression through mechanisms such as inducing oxidative stress (24), triggering chronic inflammation (25), disrupting endocrine balance, and causing DNA damage (26). Although the relationship between air pollution and respiratory diseases such as lung cancer has been widely confirmed, its association with gastric cancer (GC) still requires further investigation.

Gastric cancer (GC) represents a significant component of the global cancer burden and remains one of the most common and lethal malignancies worldwide (27). Although age (28), ethnicity (29), and family genetics (30) have been clearly recognized as risk factors for gastric cancer, environmental factors are increasingly acknowledged for their role in gastric cancer initiation and progression. Recent studies suggest that environmental exposures, including air pollution, may contribute significantly to gastric cancer development (8). However, the precise molecular mechanisms underlying the association between these environmental factors and gastric cancer have not been fully elucidated. Against this background, this study establishes a multilevel framework integrating air-pollutant-prioritized candidates with clinically relevant and spatially resolved features of GC. Above-chance overlap across complementary disease-gene definitions delineated a reproducible candidate space enriched in inflammation, hypoxia, apoptosis, metabolism and carcinogenesis. The overlap also exceeded degree- and annotation-matched null expectations, reducing the likelihood that network connectivity or annotation density alone explained the convergence. OMIM-only and TCGA-STAD differential-expression sensitivity definitions reproduced the overall enrichment direction and yielded a 23-gene cross-resource consensus set; each APRGSig gene was retained under at least two disease definitions. Retaining pollutant-specific provenance enabled the benzene/toluene branch to progress from computational prioritization to controlled AGS-cell phenotypic testing, in which both compounds increased clonogenic capacity, Matrigel traversal and wound closure. By connecting structured candidate prioritization with cross-cohort evaluation, tissue-contextualized analysis and phenotype-level evidence, this framework strengthens the biological coherence of the findings and provides a focused basis for prioritizing exposure-linked hypotheses in GC.

Within this candidate space, APRGSig condenses five complementary genes into a compact research-stage signature. The close agreement among nested, optimism-corrected and locked external C-index estimates indicates that its risk-ranking signal was preserved across cohorts, with more informative discrimination at longer horizons. Applying the fixed coefficients and cutoff without external updating further supported transferability within the evaluated external microarray cohort, and APRGSig retained an association with overall survival after adjustment for age and pathological T, N and M categories. The exploratory GC-ANN complements this result through tumor-versus-non-tumor tissue-transcriptome classification: under matched validation conditions, the five-gene nonlinear model outperformed the evaluated linear, penalized, tree-based and single-gene comparators and retained strong ranking performance on an independent platform. Together, these analyses indicate that the same small gene set carries complementary prognostic and tissue-classification information. SHAP complements these performance estimates by allocating model-specific predictive contributions among the five expression features, thereby improving model transparency.

Cell-resolved analysis extends the bulk-tissue models by defining the compartments in which the five-gene signal is expressed. HNF4A and TERT were concentrated mainly in epithelial cells, MMP9 was macrophage-enriched, and DNMT1 and GSK3B were more broadly distributed. Donor-aware scoring, UCell, leave-one-gene-out analyses and expression-matched random signatures supported a macrophage-associated multigene component. MMP9 was its largest individual contributor, but the association persisted after MMP9 exclusion, supporting interpretation of APRGSig as a cross-compartment prognostic signature with a reproducible macrophage-associated component rather than a macrophage-identity signature. Importantly, APRGSig-high macrophages and MALAT1-high macrophages represent distinct analytical concepts. APRGSig-high/low groups were score-based, specimen-specific strata used only for CellChat, whereas MALAT1-high denotes an independently reclustered, computationally defined transcriptional state. The latter was detected in all nine specimens, including primary and metastatic samples, and patient-level technical-quality measures did not differ significantly among states after multiplicity correction. Its organization remained largely stable after MALAT1 was excluded from feature selection, while donor-blocked pseudo-bulk analysis retained an IL1B/FCN1/CXCL8/NF-κB-related program. MALAT1-high is retained as a *post hoc* descriptive label and does not imply MALAT1-dependent or pollutant-specific identity. These convergent analyses support its biological interpretation as an inflammatory/stress-associated macrophage state rather than a state defined solely by MALAT1 expression.

These complementary macrophage classifications sharpen the immunological interpretation of APRGSig. Because the score strata and transcriptional states were generated independently, their outputs provide analytically distinct, complementary views of macrophage-associated biology: one centered on score-related communication and the other on inflammatory cell state. In CellChat, the score-defined APRGSig-high group showed broader predicted communication with fibroblasts, endothelial cells and epithelial cells. Predicted COLLAGEN/FN1–integrin/CD44 interactions were consistent with a macrophage–extracellular-matrix communication context, MHC-I/MHC-II with an antigen-presentation-associated context, and SPP1/CXCL signaling—including CXCL8–ACKR1—with stromal and vascular communication in the APRGSig score-based comparison. Together, these communication patterns suggest that matrix-interacting, antigen-presentation-associated and vascular-linked programs may jointly contribute to the organization of the gastric tumor microenvironment. Directionally consistent, unadjusted and section-specific positive spatial concordance between APRGSig scores and cell2location-inferred MALAT1-high abundance across four independent Visium sections provides quantitative tissue-context support. Multiplex immunofluorescence provided visual, representative and descriptive compartment-level localization evidence for APRGSig-encoded proteins within or adjacent to epithelial, macrophage-associated, endothelial and stromal regions. The convergence of donor-aware transcriptomics, communication inference, spatial statistics and protein-level compartment localization therefore extends APRGSig beyond bulk prognosis and highlights a coherent immune-microenvironmental context.

APRGSig is biologically coherent because it samples complementary GC processes rather than a single pathway. DNMT1 links maintenance methylation, inflammation-associated epigenetic change and experimentally tractable demethylation (31–35), whereas GSK3B represents context-dependent signaling across cell fate, metabolism, Wnt/β-catenin, survival and motility (36–40). HNF4A connects gastrointestinal differentiation with stress responses, tumor-associated expression, IDH1-linked metabolism and treatment response (41–45); MMP9 links extracellular-matrix remodeling to invasion, nodal involvement and survival (46–48); and TERT links telomere maintenance to proliferative capacity, apoptosis resistance and disease aggressiveness (49–53). Together, these study-supported functions place APRGSig at the intersection of tumor-cell programs, extracellular remodeling and microenvironmental regulation. The context-dependent biology of GSK3B makes it a testable candidate, while the signature’s functional breadth provides a compact framework for connecting tumor, stromal and immune processes.

The focused benzene/toluene analysis adds complementary structural, literature and functional evidence to the systems-level findings. Protocol redocking and control comparisons supported the internal reliability of the docking workflow, while the molecular-dynamics trajectories characterized numerical persistence of the formal GSK-3β–benzene and GSK-3β–toluene protein–ligand starting models. Bibliometric mapping independently placed GSK3B within established research themes involving proliferation, invasion, metastasis and treatment response. Together, these layers prioritize a testable GSK-3β-centered structural hypothesis. ShinyTHOR profiling further contextualized AGS within the heterogeneous baseline APRGSig-expression landscape of established gastric cancer cell lines. Controlled AGS-cell experiments then translated the benzene/toluene prioritization into directly observable, assay-specific phenotypes. The nonlinear CCK-8 profiles identified 2.5 mM benzene and 20 μM toluene as working conditions with sustained viability-related signals across the examined time points. At these concentrations, both compounds increased clonogenic capacity, Matrigel traversal and wound closure. Together, these biologically distinct metabolic-activity, clonogenic-capacity, Matrigel-traversal and wound-closure readouts provide a focused functional bridge between computational prioritization and gastric cancer cell behavior and establish a tractable model for subsequent molecular testing.

Taken together, the candidate, prognostic, cellular, spatial, structural and experimental layers converge on tractable molecular and immune contexts. This convergence is the principal strength of the study: it narrows a broad environmental-toxicology question to prioritized genes, defined macrophage programs and experimentally accessible GC-cell phenotypes that can guide focused mechanistic evaluation.

### 4.1 Limitations

Candidate prioritization relied on public toxicogenomic and disease-gene resources with operational thresholds, heterogeneous annotations and limited applicability to reactive or inorganic gases. Matched-null and alternative-definition analyses support overall enrichment robustness rather than independent validation of individual candidates. For NO, NO_2_, CO, SO_2_ and O_3_, prioritized genes represent exploratory downstream nodes rather than direct binding targets; the pooled union does not imply a shared mechanism. Clinical cohorts lacked exposure measurements, exposure-linked transcriptomes or histories, and tissue pollutant concentrations. APRGSig is therefore a GC prognostic signature derived from pollutant-prioritized genes, not an exposure biomarker; neither patient-level exposure–response relationships nor population-level causality was assessed.

All models were retrospective. APRGSig showed modest overall discrimination, weaker short-term performance and attenuated external calibration, while nomogram calibration was confined to the development cohort. The GC-ANN was assessed as a tissue-transcriptome classifier amid small control groups, class imbalance and platform heterogeneity, not against routine diagnostic procedures. SHAP attribution may shift among correlated genes and describes model behavior rather than biological importance, causality or pollutant responsiveness. Prospective validation and incremental clinical value remain to be evaluated. Single-cell analysis included nine specimens from six patients with unequal primary and metastatic representation, limiting site-stratified inference. MALAT1-high remains computationally defined; pseudotime and CellChat infer trajectories and communication rather than demonstrate transitions or active signaling, and APRGSig was not benchmarked against an external pan-macrophage signature. Four spatial sections were not jointly adjusted for cell-type abundance, sequencing depth or tumor domain and lacked MMP9-excluded spatial analysis. Cell2location estimates spot-level abundance rather than direct cell counts or physical proximity. Multiplex immunofluorescence in four patients provided representative compartment-level localization without whole-slide sampling, segmentation or quantitative co-expression/colocalization. These data support macrophage-state association and spatial concordance, not a composition-independent spatial mechanism.

Structural modeling examined formal benzene/toluene poses and finite 100-ns trajectories but lacked direct affinity, target engagement, phosphorylation, enzyme activity or perturbation evidence. Cellular assays used only AGS cells and one selected nominal concentration per compound after screening; no non-malignant comparator was included, and culture-medium or headspace concentrations were not measured. These conditions cannot be translated directly to human tissue exposure or generalized across cell lines or tumor-cell contexts. APRGSig genes and inflammatory mediators were not measured after exposure, and macrophage–tumor co-culture, GSK3B perturbation or rescue, mixture exposure and *in vivo* validation were not performed. CCK-8 measures metabolic activity, whereas Transwell and wound-healing assays do not establish proliferation-independent invasion or migration. Accordingly, pollutant prioritization, macrophage-state associations and AGS-cell phenotypes remain complementary evidence rather than a demonstrated causal sequence.

## 5 Conclusion

This study integrates toxicogenomic prioritization with cross-cohort modeling, single-cell and spatial analyses, structural assessment and controlled AGS-cell exposure. The resulting APRGSig provided reproducible prognostic information and showed spatial concordance with an inflammatory/stress-associated MALAT1-high macrophage state, while benzene and toluene increased AGS clonogenic capacity, Matrigel traversal and wound closure under the tested conditions. Together, these findings provide a coherent multiscale framework for prioritizing exposure-linked molecular and immune hypotheses in gastric cancer.

## Data Availability

The datasets presented in this study can be found in online repositories. The names of the repository/repositories and accession number(s) can be found in the article/**Supplementary Material**.
