# Supplementary material for: Multiscale characterization of an inflammatory macrophage state associated with an air pollutant–prioritized prognostic signature in gastric cancer
Source: Front Immunol. 2026 Sep 10;17:1951968. doi: 10.3389/fimmu.2026.1951968 (PMC13600892; doi:10.3389/fimmu.2026.1951968)
Supplement: Supplementary file 1 [file DataSheet1.docx]

**Supplementary Methods S1.** Pseudocode for nested internal validation and independent external testing of the prognostic model

This pseudocode summarizes the nested model-development and validation procedure described in Section 2.7 of the main manuscript. All data-dependent preprocessing, feature selection, hyperparameter optimization, and coefficient estimation were restricted to the corresponding outer-loop training partition.

```
Input:
    TCGA-STAD development cohort
    GSE84437 independent external validation cohort
    The 63 candidate genes entering prognostic model development
    A predefined set of 117 single- or combined-algorithm pipelines
Keep GSE84437 inaccessible throughout model development, pipeline
selection, hyperparameter optimization, coefficient estimation, and
risk-score cutoff determination.
For each candidate machine-learning pipeline:
    Initialize an empty vector for outer-loop held-out predictions.
    For each patient i in the TCGA-STAD development cohort:
        Define:
            Outer training set =
                all TCGA-STAD patients except patient i
            Outer test set =
                patient i
        Using the outer training set only:
            1. Fit all pipeline-specific preprocessing transformations.
               When centering or scaling is required, estimate the
               transformation parameters exclusively from the outer
               training set.
            2. Perform pipeline-specific feature screening.
            3. Conduct inner resampling for pipeline-specific
               hyperparameter optimization.
            4. Fit the candidate pipeline using only the outer
               training set.
            5. For the RSF–LASSO pipeline:
a. Fit the random survival forest using only the outer-training set.
b. Calculate RSF variable-importance values using only the outer-training set.
c. Retain the 15 genes with the highest training-derived RSF variable-importance values.
d. Independently fit a LASSO-penalized Cox proportional-hazards model to the candidate-gene space using only the outer-training set, with the elastic-net mixing parameter fixed at alpha = 1.
e. Select the LASSO penalty parameter by 10-fold inner cross-validation performed exclusively within the outer-training set and retain genes with nonzero coefficients at lambda.min, defined as the value yielding the minimum cross-validated partial-likelihood deviance.
f. Define the RSF–LASSO feature set as the genes retained by both the RSF and LASSO-Cox procedures.
g. Fit the fold-specific prognostic model using the RSF–LASSO feature set and the outer-training data only.
        Apply the preprocessing transformations derived from the
        outer training set unchanged to the outer test patient.
        Apply the fold-specific fitted model to the outer test patient.
        Store the resulting held-out risk prediction.
    Aggregate the held-out predictions obtained from all outer
    leave-one-out iterations.
    Calculate the out-of-fold Harrell concordance index for the
    candidate pipeline.
Rank the 117 candidate pipelines according to their out-of-fold Harrell C-indices obtained exclusively within TCGA-STAD. The prespecified parsimony rule is applied only when two or more pipelines have exactly equal calculated out-of-fold C-indices; among such tied pipelines, prioritize the pipeline retaining fewer predictors. No confidence-interval-overlap criterion, one-standard-error rule, or ΔC-index tolerance is used. This rule was defined before inspection of the 117 pipeline-performance results.
Select the optimal modeling workflow.
Refit the complete selected RSF–LASSO workflow using the full
TCGA-STAD development cohort:
```

1. Fit RSF to the candidate-gene set and calculate full-cohort variable-importance values.
2. Retain the 15 genes with the highest RSF variable-importance values.
3. Independently fit LASSO-Cox regression to the candidate-gene set with alpha = 1.
4. Optimize lambda using 10-fold cross-validation, select lambda.min, and retain genes with nonzero coefficients.
5. Define the final predictor set as the intersection of the RSF- and LASSO-prioritized genes.
6. Retain the definitive five-gene predictor set:
       DNMT1
       GSK3B
       HNF4A
       MMP9
       TERT
7. Estimate the final model coefficients using the five-gene predictor set.
8. Calculate the locked APRGSig according to:
   APRGSig =
   0.196 × Expr_DNMT1

+0.252 × Expr_GSK3B

+0.230 × Expr_HNF4A

+0.142 × Expr_MMP9

+0.305 × Expr_TERT
where Expr denotes the corresponding normalized gene-expression value on the scale used for model fitting.

9. Calculate patient-level APRGSig scores in TCGA-STAD.
10. Define the median APRGSig score in TCGA-STAD as the fixed cutoff for high- and low-risk classification.

```
The final full-cohort refit is used to determine the model intended
for external application and is not used to estimate the internally
validated out-of-fold Harrell concordance index.
Lock the following components before external evaluation:
    Preprocessing procedure
    Predictor set
    Hyperparameters
    Model coefficients
    APRGSig formula
    Risk-score cutoff
Apply the locked model once and without modification to GSE84437.
Do not use GSE84437 for:
    Preprocessing-parameter estimation
    Feature screening
    Pipeline ranking or selection
    Hyperparameter optimization
    Coefficient estimation or re-estimation
    Risk-score cutoff determination or recalibration
    Model modification
Report the external performance obtained from this single application
of the locked model to GSE84437.
Post-selection descriptive cross-cohort comparison
After completion of TCGA-STAD pipeline selection, full-cohort model refitting, model locking, and the prespecified external evaluation, cohort-specific performance estimates for the candidate pipelines were additionally summarized to characterize cross-cohort performance. These post-selection summaries were used exclusively for descriptive visualization in Fig. 3A–B and did not contribute to pipeline ranking, feature selection, hyperparameter optimization, coefficient estimation, risk-score cutoff determination, recalibration, or modification of the finalized APRGSig model.
```
