# Supplementary material for: Multiscale characterization of an inflammatory macrophage state associated with an air pollutant–prioritized prognostic signature in gastric cancer
Source: Front Immunol. 2026 Sep 10;17:1951968. doi: 10.3389/fimmu.2026.1951968 (PMC13600892; doi:10.3389/fimmu.2026.1951968)
Supplement: Supplementary file 3 [file DataSheet3.docx]

**Supplementary Figure 2**

Fig 2 . Baseline expression landscape of APRGSig genes across gastric cancer cell lines.
