# Supplementary material for: Multiscale characterization of an inflammatory macrophage state associated with an air pollutant–prioritized prognostic signature in gastric cancer
Source: Front Immunol. 2026 Sep 10;17:1951968. doi: 10.3389/fimmu.2026.1951968 (PMC13600892; doi:10.3389/fimmu.2026.1951968)
Supplement: Supplementary file 4 [file DataSheet4.docx]

**Supplementary Figure 3**

Fig 3. A. Predicted incoming and outgoing interaction strengths between APRGSig-high or APRGSig-low macrophages and each non-macrophage lineage. B. Signaling pathways showing the largest descriptive differences in aggregated communication probability between APRGSig-high and APRGSig-low macrophages. Positive values indicate higher inferred pathway activity in APRGSig-high macrophages. C. Representative ligand–receptor interactions showing the largest descriptive differences between the two macrophage groups. Blue and red points indicate APRGSig-low and APRGSig-high macrophages, respectively.
