# Supplementary material for: Multiscale characterization of an inflammatory macrophage state associated with an air pollutant–prioritized prognostic signature in gastric cancer
Source: Front Immunol. 2026 Sep 10;17:1951968. doi: 10.3389/fimmu.2026.1951968 (PMC13600892; doi:10.3389/fimmu.2026.1951968)
Supplement: Supplementary file 5 [file DataSheet5.docx]

**Supplementary Figure 4**

Fig 4. A. Dimensionality reduction and clustering of macrophages identified eight transcriptionally distinct cell clusters. B. Bubble plot depicting the association between canonical macrophage annotation genes and the eight clusters.
